# Diverse methylotrophic methanogenic archaea cause high methane emissions from seagrass meadows

**DOI:** 10.1073/pnas.2106628119

**Published:** 2022-02-14

**Authors:** Sina Schorn, Soeren Ahmerkamp, Emma Bullock, Miriam Weber, Christian Lott, Manuel Liebeke, Gaute Lavik, Marcel M. M. Kuypers, Jon S. Graf, Jana Milucka

**Affiliations:** ^a^Department of Biogeochemistry, Max Planck Institute for Marine Microbiology, 28359 Bremen, Germany;; ^b^HYDRA Marine Sciences GmbH, 77815 Bühl, Germany;; ^c^Symbiosis Department, Max Planck Institute for Marine Microbiology, 28359 Bremen, Germany

**Keywords:** methane, methanogenesis, seagrass, archaea, blue carbon

## Abstract

Marine coastlines colonized by seagrasses are a net source of methane to the atmosphere. However, methane emissions from these environments are still poorly constrained, and the underlying processes and responsible microorganisms remain largely unknown. Here, we investigated methane turnover in seagrass meadows of *Posidonia oceanica* in the Mediterranean Sea. The underlying sediments exhibited median net fluxes of methane into the water column of ca. 106 µmol CH_4_ ⋅ m^−2^ ⋅ d^−1^. Our data show that this methane production was sustained by methylated compounds produced by the plant, rather than by fermentation of buried organic carbon. Interestingly, methane production was maintained long after the living plant died off, likely due to the persistence of methylated compounds, such as choline, betaines, and dimethylsulfoniopropionate, in detached plant leaves and rhizomes. We recovered multiple *mcrA* gene sequences, encoding for methyl-coenzyme M reductase (Mcr), the key methanogenic enzyme, from the seagrass sediments. Most retrieved *mcrA* gene sequences were affiliated with a clade of divergent Mcr and belonged to the uncultured *Candidatus* Helarchaeota of the Asgard superphylum, suggesting a possible involvement of these divergent Mcr in methane metabolism. Taken together, our findings identify the mechanisms controlling methane emissions from these important blue carbon ecosystems.

## Significance

Seagrass meadows colonize shallow coastlines around the world and represent sites of intense carbon cycling. Due to their capacity to produce methane, seagrass ecosystems constitute net sources of methane to the atmosphere. Here, we identify key processes and microorganisms responsible for methane formation in seagrass-covered sediments in the Mediterranean Sea. Our work shows that methane is solely formed from methylated compounds that are produced and released by the plant itself. Due to the persistence of these compounds in buried plant material, microbial methane production continues long after the death of the living plant. These results provide a comprehensive understanding of methane production in seagrass habitats, thereby contributing to our knowledge on these important blue carbon ecosystems.

About one-half of global methane emissions [ca. 270 Tg CH_4_ ⋅ yr^−1^ (1)] stem from aquatic environments, mainly the inland waters. In the marine environment, coastal areas represent methane hotspots, releasing around 8 to 13 Tg CH_4_ ⋅ yr^−1^ (1, 2) thus highly exceeding emissions from the open ocean [0.6 to 1.2 Tg CH_4_ ⋅ yr^−1^ (3)]. High methane emissions from coastal regions are caused by high fluxes of methane from the sediment, of which more than two-thirds is biogenic in origin [i.e., produced by a microbial process called methanogenesis (4, 5)]. Methanogenesis is a form of anaerobic respiration during which oxidized carbon is used as the terminal electron acceptor. The biochemical pathway of methanogenesis contains a conserved set of enzymes (6) of which methyl-coenzyme M reductase (Mcr) is the key one. Therefore, the gene encoding for Mcr (*mcrA*) is generally used as a universal phylogenetic marker to identify methanogenic microorganisms.

The capacity for methanogenesis is constrained to a group of strictly anaerobic methanogenic archaea (7, 8). Until recently, methanogens were believed to belong exclusively to the phylum Euryarchaeota, and to date, most described methanogens still affiliate with this phylum. However, several recent studies have described novel putatively methanogenic archaeal phyla based on the presence of the *mcrA* gene in their genomes. These novel putative methanogens belong to *Candidatus* Bathyarchaeota (9), *Candidatus* Methanomethyliaceae [formerly Verstraetarchaeota (10)], and Thermoplasmata (11). These observations suggest that the metabolic trait of methanogenesis is phylogenetically more widespread than previously thought. Additionally, genes encoding putative methyl-CoM reductase–like enzymes have also been found in the genomes of *Candidatus* Syntrophoarchaeum (12) and *Candidatus* Helarchaeota (13), albeit here this enzyme is presumed to be involved in the anaerobic oxidation of butane.

Due to their obligately anaerobic nature, methanogens are typically constrained to permanently anoxic habitats where other, thermodynamically more favorable electron acceptors are absent. Methanogens have a very limited substrate range and can only use a handful of simple organic compounds. Depending on the utilized substrate, the pathways of methanogenesis classify as hydrogenotrophic (use H_2_ to reduce CO_2_ to CH_4_), acetoclastic (use acetate disproportionation to form CH_4_), and methylotrophic (use methyl groups of methylated compounds, such as methylamine, to form CH_4_).

Hydrogenotrophic methanogenesis is the predominant pathway of methane production in anoxic marine sediments, followed by acetate disproportionation (14). Both hydrogen and acetate are formed in situ mainly through the activity of fermentative bacteria. Additionally, plant-associated fungi can facilitate plant tissue breakdown (15), thereby providing methanogenic substrates to the microbial community. However, hydrogen and acetate can also be used by sulfate-reducing bacteria (SRB) and, in fact, SRB routinely outcompete methanogens for these compounds (16, 17). Therefore, hydrogenotrophic and acetoclastic methanogenesis usually do not co-occur with sulfate reduction in marine sediments (7, 16, 18), and H_2_ and acetate are thus also referred to as so-called competitive substrates (19).

In contrast, methylated compounds such as methylated amines (mono-, di-, and trimethylamine) or methylated sulfides (dimethylsulfide; DMS) can exclusively be used by methanogens and represent so-called noncompetitive substrates (20). Methylotrophic methanogenesis can therefore readily proceed under high ambient sulfate concentrations and has been reported to dominate in, for example, organic-rich muddy sediments (21, 22) and hypersaline environments (23). Methylated compounds, such as betaines and methylamines, are ubiquitous in coastal marine environments (24, 25) as different marine organisms (including marine plants) produce them to cope with osmotic stress (26). The degradation of these compounds can lead to the release of methane, and methylotrophic methanogenesis is thus expected to play an important role in coastal sediments with high salinity and under the influence of algae or plants (18, 20).

Many shallow coastlines are vegetated by macroalgae and seagrasses (27). Seagrasses are marine flowering plants that form some of the most productive ecosystems on Earth (28). At the same time, seagrass ecosystems, such as sediments colonized by eelgrass (*Zoster* sp.) and turtlegrass (*Thalassia* sp.) are recognized as important sources of methane to the atmosphere (29–31) with estimated emissions ranging from about 0.1 to 2.7 Tg CH_4_ ⋅ yr^−1^ (1, 31, 32). Methane emissions from seagrass ecosystems are somewhat lower than from mangroves and salt marshes, but seagrasses cover more coastal areas than mangroves and marshes combined (32). *Posidonia* seagrasses can be found throughout the Mediterranean Sea (*Posidonia oceanica*) and around the southern coast of Australia (*Posidonia australis*). Due to their large size and their capacity to form massive underground peat deposits (33), *Posidonia* meadows represent an important marine blue carbon ecosystem (34, 35). To date, there are no reported methane production rates for *Posidonia* seagrasses, and the metabolic processes and microorganisms involved in methane metabolism in seagrass ecosystems are still largely unknown.

In this study, we describe the mechanism by which methane emissions are sustained from sediments underlying living and dead seagrass meadows of *P. oceanica* in the Mediterranean Sea, and we quantify the efficiency of the biological methane filter in these sediments. We also present a comprehensive analysis of the microbial community associated with these important coastal habitats, with a particular focus on the diversity of methanogenic microorganisms.

## Results

### Porewater Chemistry and Methane Production of Sediments Underlying *P. oceanica* Meadows.

**Fig. 1.**
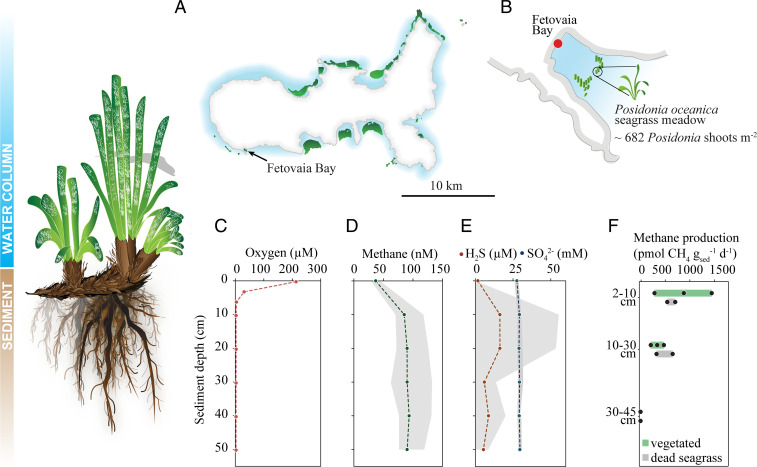
Characteristics of sediments underlying *P. oceanica* seagrass meadows in Fetovaia Bay. Illustration of leaf, rhizome, and root growth of *P. oceanica*; roots and dead plant material accumulate as thick peat layers in the sediment. CO_2_ fixation takes place in the plant’s leaves, and organic carbon is transported and stored in the plant's rhizomes and roots. (*A*) Map of the island of Elba (Italy) with *P. oceanica* distribution (highlighted in green) after ref. 38; note that the distribution of *P. oceanica* along the coast is not comprehensively documented. (*B*) Sketch of Fetovaia Bay; the investigated seagrass patch had a density of ∼680 *P. oceanica* shoots per m^2^. Next to the seagrass meadow, a small patch was located where the living plants died off ca. 25 y ago. Porewater profiles of (*C*) oxygen, (*D*) methane, (*E*) sulfide (red), and sulfate (blue). The dotted lines depict average concentrations measured from four replicate porewater profiles, and the gray shaded area represents the concentration range. (*F*) Methane production rates in sediment incubations from different depth layers in vegetated and dead seagrass sediment.

We sampled sediments underlying living and dead *P. oceanica* seagrass meadows in the bay of Fetovaia, Elba (Italy) on three campaigns over the course of two consecutive years (Fig. 1 *A* and *B*). We investigated sediments colonized by living *Posidonia* seagrasses (hereafter referred to as “vegetated sediment”) as well as sediments underneath dead *Posidonia* meadows (hereafter referred to as “dead seagrass sediment”). Both types of sediments consisted of fine sand (*SI Appendix*, Fig. S2 and Text S1). On each campaign, the porewater was collected from distinct depth horizons using gas-tight stainless-steel porewater lances (*Materials and Methods*). Oxygen concentrations were below detection levels (ca. 1 µM) in all but the uppermost depth horizon (0- to 6-cm sediment depth, Fig. 1*C*). Porewater sulfide concentrations were persistently very low (below 0.05 mM) and did not follow a clear depth trend. Sulfate concentrations were constant down to ca. 50-cm depth, at a concentration of around 29 mM with small fluctuations (Fig. 1*E*). Porewater methane concentrations increased from 37 ± 7 nM in surface sediment to 85 ± 21 nM at 10-cm depth and then remained stable down to a sediment depth of 50 cm (Fig. 1*D*).

To quantify the methane flux from the sediment into the water column, we incubated whole sediment cores from the vegetated sediment (*n* = 11) in the dark at ambient temperature (22 °C). Diffusive methane fluxes from these replicate cores varied from 5 to 490 µmol ⋅ m^−2^ ⋅ d^−1^ with an overall median of 106 µmol ⋅ m^−2^ ⋅ d^−1^ over a 24-h time period (*SI Appendix*, Fig. S7). Some of the incubated cores (*n* = 7) contained an intact seagrass plant, and the methane fluxes from vegetated cores with a plant were significantly higher than fluxes from vegetated cores without a plant (one-way ANOVA, *P* = 0.037; see *Materials and Methods* for details on core composition and *SI Appendix*, Fig. S7).

To investigate the potential of vegetated sediments to produce methane, we set up unamended incubations with sediment from three different depth horizons. The depth horizons were defined visually, based on the sediment composition. The uppermost surface layer was sandy, with plant rhizomes and roots (D1, 2 to 10 cm), the intermediate layer contained a thick mesh of plant roots (D2, 10 to 30 cm), and the deepest layer consisted of highly degraded plant material (D3, 30 to 45 cm; *Materials and Methods*). Highest methane production rates were observed in the uppermost sediment layer (2 to 10 cm) with an average net rate of 887 pmol ⋅ g_sed_^−1^ ⋅ d^−1^ (Fig. 1*F*). At 10- to 30-cm depth, methane production proceeded at a lower rate of 346 pmol ⋅ g_sed_^−1^ ⋅ d^−1^, and no methane production was detected in sediment incubations from the deepest layer (30 to 45 cm). When integrating the volumetric rates over the incubated sediment depth, these rates amount to a net methane flux of ca. 113 µmol ⋅ m^−2^ ⋅ d^−1^.

In addition to vegetated sediments, porewater was also collected in dead seagrass sediments directly adjacent to the seagrass patch. Here, the *Posidonia* plants died off more than 25 y ago, leaving behind dead seagrass sediments overlying buried peat deposits (*SI Appendix*, Fig. S3*A* and Text S3). The methane concentration in the porewater was notably higher and showed a pronounced peak in deeper sediment layers (*SI Appendix*, Fig. S3*B*), coinciding with a simultaneous increase in sulfide concentrations (*SI Appendix*, Fig. S3*C*). Sulfide concentrations reached up to 5 mM between 20- and 40-cm sediment depths, whereas sulfate concentrations remained constant around 29 mM (*SI Appendix*, Fig. S3*C*). Unamended incubations of these dead seagrass sediments showed methane production occurring in the two upper depths (638 and 490 pmol ⋅ g_sed_^−1^ ⋅ d^−1^) with rates largely similar to the rates measured in incubations of vegetated sediment. No methane production rates could be detected in the incubations from the deepest depth (Fig. 1*F*). When integrating the volumetric methane production rates over the incubated sediment depth, these rates amount to a net methane flux of ca. 116 µmol ⋅ m^−2^ ⋅ d^−1^. For comparison, direct methane flux measurements from incubated intact dead seagrass sediment cores (*n* = 4) over a 24-h time period amounted to a median flux of 142 µmol ⋅ m^−2^ ⋅ d^−1^.

### Pathways of Methane Production in Vegetated Sediments.

**Fig. 2.**
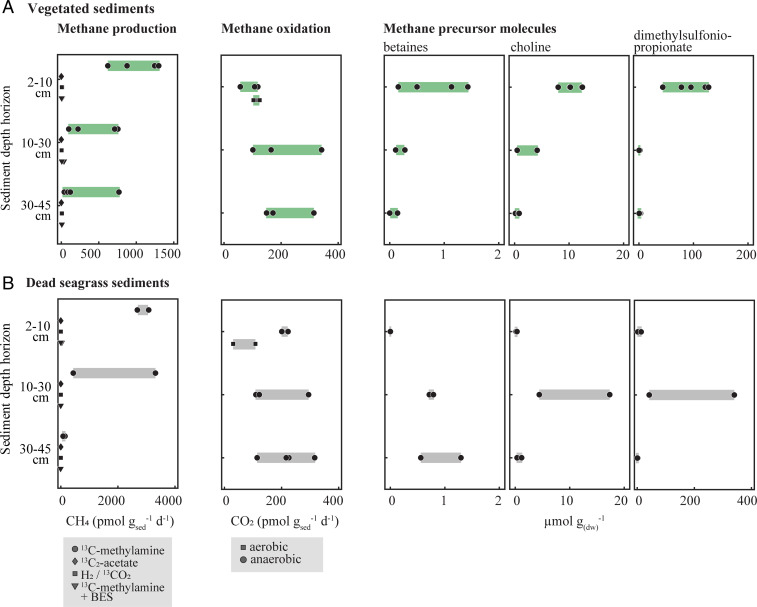
Rates of methane production and oxidation and the distribution of methane precursor molecules in living (*A*) and dead (*B*) seagrass sediments. Sediment incubations were performed to test for methane production from ^13^C_2_-acetate, ^13^C-bicarbonate (added with H_2_), ^13^C-monomethylamine, and ^13^C-monomethylamine added together with BES, a methanogenesis inhibitor (*Left*). Methane oxidation activity was tested under aerobic (only surface sediment layer, square symbol) and anaerobic conditions (all three depths, circles) (*Middle*). The abundance of three putative methane precursor molecules (betaines, choline, and DMSP) was analyzed in seagrass rhizomes collected from the same sediment horizons as used for the incubation experiments (*Right*).

In order to investigate which pathway of methane production was active in vegetated sediments, sediment and peat from three depth horizons (2 to 10, 10 to 30, and 30 to 45 cm) were incubated with various ^13^C-labeled substrates to test for the presence of acetoclastic, hydrogenotrophic, and methylotrophic methanogenesis. The increase of ^13^C-labeled methane in the incubations over time was used to calculate methane production rates. Thus, all reported rates represent net turnover rates of the added tracer and assume an equal background concentration of the nonlabeled substrate across incubations and depths. Methane production from acetate and H_2_/CO_2_ was undetectable in all three sampling campaigns and in all investigated depths (Fig. 2 *A* and *B*). Only incubations with added methylated substrates showed methane production (Fig. 2). Incubations from the uppermost sediment layer (2 to 10 cm) generally yielded the highest methane production rates averaging 1,017 pmol ⋅ g_sed_^−1^ ⋅ d^−1^. The rates then tend to decrease with depth (453 pmol ⋅ g_sed_^−1^ ⋅ d^−1^ at 10 to 30 cm and 260 pmol ⋅ g_sed_^−1^ ⋅ d^−1^ at 30 to 45 cm). Near complete inhibition of methane production was observed when 10 mM 2-bromoethanesulfonate (BES; a competitive inhibitor of archaeal methanogenesis) was added to methylamine-supplemented incubations.

Other methylated compounds, such as dimethylamine (DMA), DMS, and methanol also stimulated methane production in vegetated sediment incubations (*SI Appendix*, Fig. S4*A*). In general, the methane production rates from dimethylated compounds (DMA and DMS) were roughly twice as high (607 and 580 pmol ⋅ g_sed_^−1^ ⋅ d^−1^) as in incubations with compounds carrying a single methyl group (MMA; 260 pmol ⋅ g_sed_^−1^ ⋅ d^−1^). Incubations with sediment from the same core showed similar degree of activity with different substrates (i.e., the highest rates for DMA were measured in incubations from the same core for which there were highest rates for MMA and DMS as well). For all tested substrates, methane concentration increased at a linear or slightly exponential rate. Exponential rates might result from either diffusion limitation of the labeled substrate at the start of the experiment, heterogeneous substrate distribution, or actual microbial growth during the 24-h incubation (*SI Appendix*, Fig. S4 *B* and *C*).

Incubations of dead seagrass sediment also only showed methane production from methylated compounds. Rates of methane production from monomethylamine in the two uppermost sediment horizons (2 to 10 and 10 to 30 cm) were about twice as high as in incubations of vegetated sediment from the same depth. In contrast, the rates in the deepest sediment horizon (30 to 45 cm) were considerably lower for incubations supplemented with monomethylamine and other methylated compounds (*SI Appendix*, Fig. S4*C*).

### Aerobic and Anaerobic Methane Oxidation in Vegetated Sediments.

The potential for microbial methane oxidation was tested in sediment incubations from October 2018 and September 2019. For this, slurries containing sediment from three depth horizons were supplemented with ^13^CH_4_, and methane oxidation was monitored as the production of ^13^CO_2_ over 24 h. Methane oxidation experiments were set up under both anoxic (all three depths) and oxic conditions (only surface 2 to 10 cm) (Fig. 2*A*). Oxic incubations (around 3.5 ± 2 µM O_2_) were repeatedly replenished with oxygenated water throughout the course of the incubation. The low micromolar oxygen concentrations in these incubations were chosen in order to mimic the in situ conditions with low oxygen concentrations but constant flux.

Of the two investigated campaigns, methane oxidation was only detected in incubations from October 2018, whereas no methane oxidation rates could be detected in incubations from September 2019 (detection limit ca. 60 pmol ⋅ g_sed_^−1^ ⋅ d^−1^). In the vegetated sediment, the rates of aerobic methane oxidation reached around 114 ± 10 pmol ⋅ g_sed_^−1^ ⋅ d^−1^ in the surface layer. Anaerobic methane oxidation rates in the surface layer were somewhat lower (94 ± 33 pmol ⋅ g_sed_^−1^ ⋅ d^−1^), but they increased with sediment depth (from 202 ± 125 pmol ⋅ g_sed_^−1^ ⋅ d^−1^ at 10 to 30 cm to 212 ± 90 pmol ⋅ g_sed_^−1^ ⋅ d^−1^ at 30 to 45 cm).

In incubations of dead seagrass sediment (Fig. 2*B*), rates of aerobic methane oxidation in the surface sediment layer (2 to 10 cm) were lower (71 ± 55 pmol ⋅ g_sed_^−1^ ⋅ d^−1^) in comparison to incubations from vegetated sediment. Rates of anaerobic methane oxidation remained similar over the three sediment depths (D1: 212 ± 15 pmol ⋅ g_sed_^−1^ ⋅ d^−1^, D2: 189 ± 88 pmol ⋅ g_sed_^−1^ ⋅ d^−1^, D3: 218 ± 82 pmol ⋅ g_sed_^−1^ ⋅ d^−1^) and were in the same range as those in vegetated sediment incubations.

### Abundance of Methylated Compounds in Plant Tissue and Their Utilization.

To identify a potential source of methanogenic substrates in the *Posidonia*-covered sediments, we analyzed living and dead rhizomes and leaves for the presence of methylated substrates that could act as methane precursors. The rhizomes were collected from the same depth horizons that were used for the incubation experiments; the analyzed seagrass leaves were cut off living plants. Various methylated compounds known to act as direct or indirect methane precursors (betaines, such as glycinebetaine and prolinebetaine, choline, and dimethylsulfoniopropionate [DMSP]), were detected in seagrass rhizomes collected from all three depth horizons (Fig. 2). The abundance of these compounds changed with depth, with rhizomes retrieved from the uppermost depth horizon containing highest amounts of the investigated compounds. Interestingly, this distribution reflected the observed trend in the measured methane production rates, with highest methane production rates in surface sediments coinciding with the highest concentration of methylated compounds in seagrass rhizomes.

In dead seagrass sediments, the collected rhizomes visually appeared to be more degraded than rhizomes collected from vegetated sediments. However, the concentrations of betaines, choline, and DMSP in these rhizomes were in a similar range, albeit they showed the opposite trend with depth; that is their concentrations increased slightly with depth. In the surface layer of dead seagrass sediments, the concentrations of betaines, choline, and DMSP in rhizomes were markedly lower than in the neighboring vegetated sediments (Fig. 2).

Of all investigated plant parts, the highest concentrations of choline and DMSP were detected in seagrass leaves (10 µmol choline ⋅ g_(dw)_^−1^ and 180 µmol DMSP ⋅ g_(dw)_^−1^; Fig. 2 and *SI Appendix*, Table S3). For comparison, the concentrations of betaines were comparable to those found in buried rhizomes.

To test for a potential link between these plant-derived methylated compounds and methane production, we set up sediment incubations supplemented with unlabeled glycinebetaine. Glycinebetaine is a major marine plant osmolyte and was found to be abundant in all investigated *P. oceanica* plant parts. In the amended incubations, the onset of methane production was immediate and proceeded linearly over the course of 24 h (*SI Appendix*, Fig. S5*B*). For comparison, unamended sediment incubations from the same sediment depth horizon (30 to 45 cm) did not show any significant methane production (*SI Appendix*, Fig. S5*A*).

### Microbial Community Composition.

We analyzed the microbial community composition in all three depth horizons of three vegetated seagrass cores that had been used to measure methane production rates. First, the 16S ribosomal RNA (16S rRNA) gene sequences from unassembled metagenomes were taxonomically classified. In all investigated samples (*n* = 9), the microbial community was dominated by bacterial 16S rRNA gene sequences. The relative abundance of archaeal 16S rRNA gene sequences increased with depth, from ∼6% in the uppermost sediment layer between 2 and 10 cm, to ∼12% in the sediment between 10 and 30 cm and ∼16% in the sediment between 30 and 45 cm. It should be noted that archaeal abundances in the presented dataset might be underestimated, as bacteria, in contrast to archaea, tend to contain multiple copies of the 16S rRNA gene per genome (36).

**Fig. 3.**
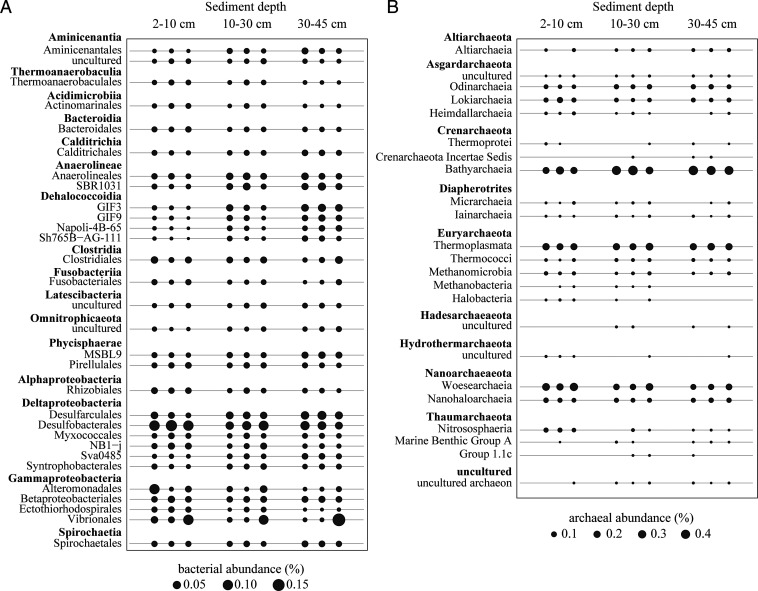
Microbial diversity in sediments underlying seagrass meadows of *P. oceanica* at different depths. (*A*) Relative abundance of the 30 most abundant bacterial taxa grouped at order level based on 16S rRNA gene abundances. (*B*) Relative abundance of archaeal 16S rRNA gene sequences grouped at phylum level.

Overall, most bacterial 16S rRNA gene sequences in surface (D1, 2 to 10 cm), middle (D2, 10 to 30 cm), and deep (D3, 30 to 45 cm) sediment layers were classified as Gammaproteobacteria (27, 21, and 12%, in D1, D2, and D3, respectively; percentages give the average relative abundance to all bacterial sequences) and Deltaproteobacteria (21, 20, and 18%); the most abundant families of the latter included *Desulfarculaceae* and *Desulfobacteraceae*. Other obligate anaerobic taxa (e.g., *Anaerolinea* and *Dehalococcoidia*) also increased in abundance towards deeper sediment depths (Fig. 3*A*). Taxa belonging to putative fermenters, such as Clostridiales and Vibrionales, were stable members of the community in all depths.

Most recovered archaeal 16S rRNA gene sequences in surface, middle, and deep sediment layers belonged to Woesearchaeia (25, 17, and 13% in D1, D2, and D3, respectively), Nanohaloarchaeia (7, 6, and 7%), Bathyarchaeia (22, 36, and 40%), Lokiarchaeia (8, 4, and 5%), Odinarchaeia (4, 7, and 6%), Thermoplasmata (19, 21, and 21%), and Nitrososphaeria (5, 1, and 0%) (Fig. 3*B*; percentages give relative abundance to all archaeal sequences averaged per depth). Some taxonomic groups showed a clear depth distribution [e.g., aerobic Nitrososphaeria were only detected in the surface sediment horizon and only in low abundances in deeper layers (below 10 cm, Fig. 3*B*)]. Similarly, the relative abundance of Woesearchaeia sequences decreased with depth, whereas Bathyarchaeia sequences increased with depth (Fig. 3*B*).

Few of the retrieved 16S rRNA gene sequences were affiliated with classical methanogenic orders within the phylum of Euryarchaeota, such as Methanofastidiosales, Methanomicrobiales, Methanosarcinales, and Methanobacteriales. Among those, members of the *Methanococcoides* had the highest relative abundance in all three sediment horizons and showed a decreasing trend with depth. Overall, the relative abundances of these “classical” euryarchaeal methanogenic taxa was low (< 2% of all archaeal 16S rRNA sequences) and likewise decreased with depth.

Interestingly, taxa belonging to novel putatively methanogenic orders were overrepresented in the archaeal community of vegetated sediments. Up to 40% of archaeal 16S rRNA gene sequences belonged to the candidate phylum Bathyarchaeota. In order to investigate the diversity of Bathyarchaeota in more detail, 16S rRNA gene sequences were assembled from metagenomic reads and used for phylogenetic analyses. The bathyarchaeal 16S rRNA sequences affiliated with five different subgroups of Bathyarchaeota [formerly MCG (37); *SI Appendix*, Fig. S6], mostly within the subgroups MCG-6 and MCG-8. The MCG-8 group was previously shown to contain a sequence belonging to a putative methanogen/alkane oxidizer (BA2) encoding the *mcrA* gene (9). Our retrieved sequences formed a distinct subcluster within the MCG-8 group (*SI Appendix*, Fig. S6).

Other putatively methanogenic archaeal lineages, such as Thermoplasmata and Methanomassiliicoccales, were also present in the vegetated sediment, at relative abundances of up to 21% and <1%, respectively. However, most sequences that were classified as Thermoplasmata affiliated with Marine benthic group D and DHVEG-1, for which no anaerobic hydrocarbon metabolism has been proposed so far.

We also searched for putative aerobic and anaerobic methane-oxidizing bacteria and archaea in the sample. Known aerobic gammaproteobacterial methanotrophs of the order *Methylococcales*, anaerobic bacterial methanotrophs of the phylum NC10, as well as anaerobic archaeal methanotrophs (ANME-1, -2, -3) were detected in most samples but only at very low abundances (max. 0.005% of bacterial or archaeal sequences). The abundance of aerobic and anaerobic methanotrophs suggests that potential methane oxidation in these sediments might be coupled to the reduction of oxygen and sulfate in the oxic and anoxic parts of the sediment, respectively. Additionally, anaerobic methane oxidizers could also be active in anoxic microniches in oxic sediment depths. The strikingly low abundance of archaeal and bacterial methanotrophs in the sedimentary microbial community fits well to the measured low rates of both aerobic and anaerobic methane oxidation (Fig. 2) and could be explained by the low methane concentrations in the sediment.

### Methyl-Coenzyme M Reductase Subunit A Phylogeny.

**Fig. 4.**
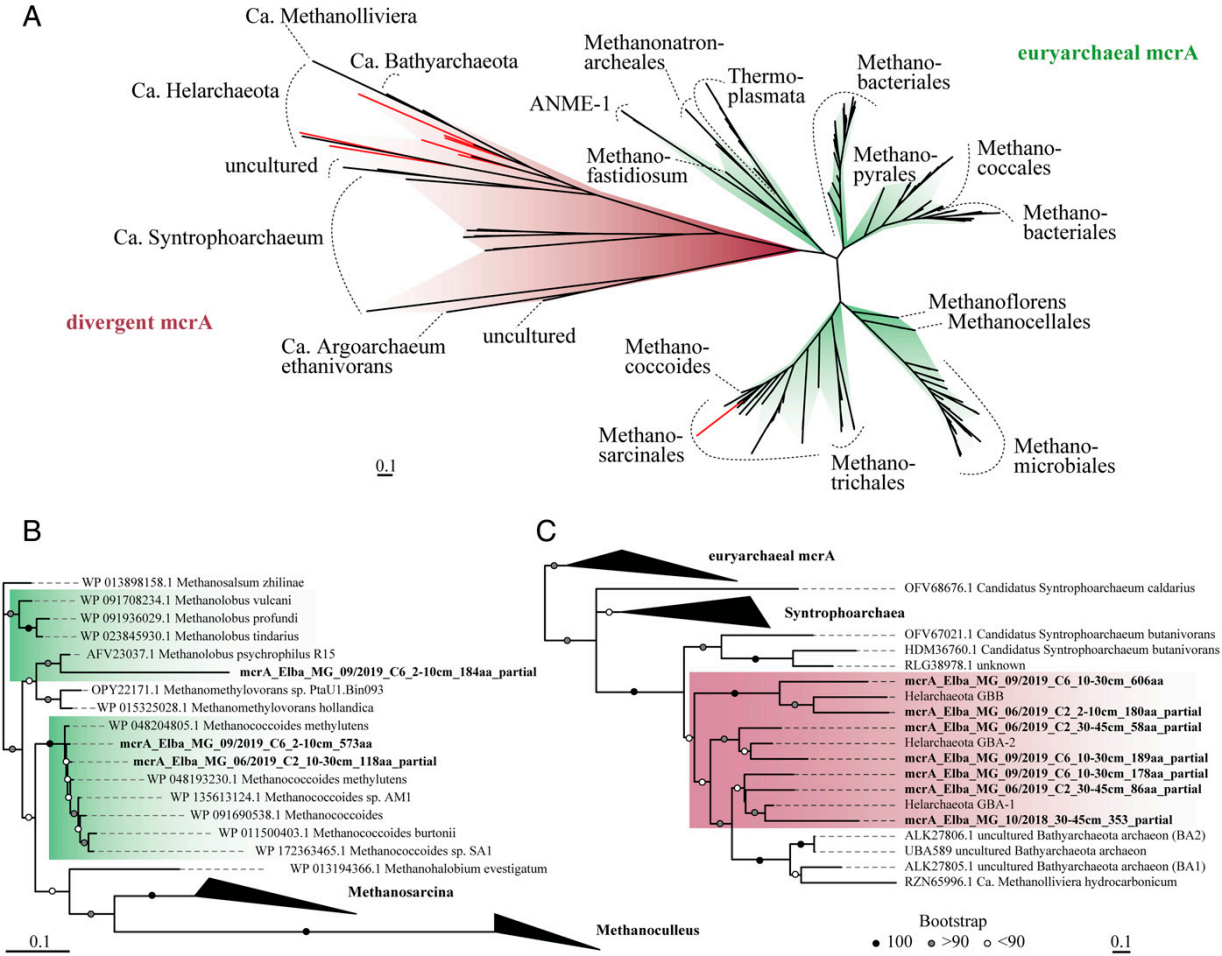
Phylogeny of McrA sequences recovered from vegetated sediments. (*A*) Maximum likelihood tree of 214 McrA protein sequences of classical euryarchaeal as well as divergent McrA protein sequences obtained from GenBank, together with 10 McrA protein sequences recovered from our sediment metagenomes (highlighted in red). (*B*) Maximum likelihood tree of 34 McrA protein sequences belonging to the order Methanosarcinales showing the placement of the metagenome-recovered McrA protein sequence (highlighted in bold) within a cluster of *Methanococcoides* sequences. (*C*) Maximum likelihood tree showing the placement of seven McrA protein sequences from vegetated sediments (highlighted in bold) in a subcluster together with divergent McrA sequences. Bootstrap support was generated from 1,000 iterations and is represented as black, gray, and white dots for 100, >90, and <90% support, respectively. Scale bars indicate substitutions per site.

We also searched for sequences of the methyl-coenzyme M reductase A gene (*mcrA*), the marker gene for methanogenic and methanotrophic archaea, and we phylogenetically analyzed McrA protein sequences retrieved from assembled metagenomes from vegetated sediment (Fig. 4*A*). From the surface sediment layer, we recovered two McrA sequences. One McrA sequence was almost complete in length (573 amino acids) and was most similar to McrA sequences belonging to *Methanococcoides*. Phylogenetic analysis showed the placement of the recovered McrA sequence within a cluster of Methanosarcinales McrA sequences and affiliated closest with a sequence of *Methanococcoides methylutens* (WP_048204805.1, 98.6% amino acid identity) (Fig. 4*B*). The second McrA sequence was only partial (184 amino acids) and was most similar to *Methanolobus*, which is closely affiliated with the Methanosarcinales. Another McrA sequence that affiliated with the Methanosarcinales sequences was recovered from sediments between 10- and 30-cm depth.

Additionally, we recovered seven McrA protein sequences that clustered with the group of “divergent” McrA sequences composed of *Ca.* Helarchaeota, *Ca.* Bathyarchaeota, and *Ca.* Syntrophoarchaeum (Fig. 4*C*). One of these divergent McrA sequences was near full length (606 amino acids); the others were of partial length (ranging from 58 to 353 amino acids in length). All recovered McrA sequences that clustered together with divergent McrA sequences were closest affiliated to McrA sequences from the candidate phylum Helarchaeota of the Asgard archaea (Fig. 4*C*).

## Discussion

### Methane Fluxes from *P. oceanica* Seagrass Meadows.

*P. oceanica* is the dominant seagrass species in the Mediterranean Sea and covers at least 11,687 km^2^ of its coastline (38) (*SI Appendix*, Fig. S2*A*). We show that the *P. oceanica*–covered sediments off the island of Elba were a net source of methane to the water column. The measured net flux of methane from whole-core incubations was comparable to the flux from depth-integrated methane production rate measurements in nonamended sediment incubations; however, the fluxes varied strongly between the investigated cores (4.6 to 490 µmol ⋅ m^−2^ ⋅ d^−1^). It is notable that the methane emissions into the water column and the methane-producing potential of the dead seagrass sediment remained largely comparable to that of the living seagrass meadow (median of 142 µmol ⋅ m^−2^ ⋅ d^−1^ versus 106 µmol ⋅ m^−2^ ⋅ d^−1^, respectively; *SI Appendix*, Fig. S7). This suggests that methane production can be sustained in seagrass sediments long after the living plant’s disappearance. We propose that this observation can be explained by the persistence of methylated compounds in the plant’s tissue, as for example choline, betaines and DMSP were found in high concentrations also in partially degraded rhizomes and leaves buried in or lying on top of the dead seagrass sediments (Fig. 2 and *SI Appendix*, Table S3). It should be noted that our measured methane fluxes represent dark fluxes and do not account for potential changes associated with the plant’s diel cycle. However, current reports suggest that methane emissions from seagrass-covered sediments might be independent of light or dark conditions (30, 31). Although these fluxes represent net fluxes that account for methane oxidation, the gross methane fluxes are likely not substantially different because the rates of methane oxidation in these sediments were lower than the methanogenesis rates. This indicates that the microbial methane filter in these sediments is rather inefficient and cannot substantially mitigate methane emissions into the water column.

Our measured methane fluxes from *P. oceanica* meadows (upscaled 0.0003 to 0.033 Tg CH_4_ ⋅ yr^−1^ for the Mediterranean Sea) are among the highest reported to date from seagrass meadows (30, 31, 39). Methane appears to be efficiently transported out of the sediment through advective and/or plant-mediated transport (*SI Appendix*, Texts S1 and S3) as indicated by the low methane concentrations in the vegetated sediment (despite high methane production rates) and the lack of structure in the methane concentration profile. Our flux measurements captured the diffusive transport of methane from the sediment but did not account for advective and plant-mediated transport processes or the effects of waves and water movement on methane exchange between the sediment and the water column. Therefore, our measurements may underestimate the total methane emissions from *Posidonia*-covered sediments. Due to the shallow water depths of the seagrass beds (as a consequence of their dependence on light) and the generally low methane oxidation rates in the mixed water column (40), the emitted methane will likely efficiently exchange between the water column and the atmosphere, similar to other settings (41). It has been pointed out previously that the methane emissions from seagrass-covered sediments into the water column partially offset the effect of CO_2_ uptake by the plant and therefore affect the blue carbon function of these ecosystems (see discussion in *SI Appendix*, Text S2).

### Microbial Processes Underlying Methane Emissions from *P. oceanica* Meadows.

The ability of seagrass ecosystems to produce methane is typically assigned to their capacity to release high amounts of labile organic carbon into the underlying sediments (31). Through their degradation, methanogenic substrates such as hydrogen and acetate can be produced. *Posidonia* seagrasses additionally bury large amounts of plant material in the form of massive underground peat deposits (33) (*SI Appendix*, Fig. S3*A*) a feature analogous to terrestrial peatlands, another recognized source of methane to the atmosphere (42). In terrestrial peats, the predominant modes of methane production are acetoclastic and hydrogenotrophic methanogenesis (43). On the contrary, our substrate-addition experiments with *P. oceanica*–covered sediments showed that no methane was produced from competitive substrates, such as hydrogen or acetate, as these substrates were likely used up by the abundant sulfate-reducing Deltaproteobacteria (Fig. 3). This highlights an interesting difference between microbial methane production in marine and terrestrial peat ecosystems.

Methylotrophic methanogenesis was the sole detected pathway of methane production in vegetated as well as in dead seagrass sediments (Fig. 2). This agrees with observations from other vegetated marine sediments, such as salt marshes (20) and intertidal sediment containing algal detritus (18, 44), that methanogenesis is mainly fueled by noncompetitive substrates, which are largely inaccessible to sulfate reducers.

Highest rates of methane production were consistently measured in the uppermost sediment horizon, and the rates appeared to decrease in deeper depths. However, it should be noted that all rates represent turnover rates of the added tracer and not total turnover rates (i.e., do not account for nonlabeled substrates present in the sediment; *SI Appendix*, Text S4). Importantly, all measured activity could clearly be attributed to archaeal methanogenesis as the addition of BES, a specific inhibitor of the archaeal methanogenic enzyme Mcr, consistently inhibited methane production (Fig. 2). Our analyses showed that the plant rhizomes in the uppermost surface layer contained the highest concentrations of methylated compounds; additionally, the surface layer likely receives a continuous supply of these methane precursors from leaf debris. At the same time, the surface sediments also experience periodic events of oxygenation (Fig. 1*C*), which can potentially interfere with the strictly anaerobic process of archaeal methanogenesis. However, the phenomenon of oxygen-tolerant methane production via methanogenesis has previously been observed in, for example, soils where methanogens seem to be able to sustain their activity through the expression of genes controlling oxygen toxicity (e.g., catalases) (45) or by occupying anoxic microniches of the oxygenated soil or sediment layer (46).

Seagrasses are a rich source of small, methylated compounds that can act as direct or indirect methane precursors. For example, methanol may be formed during bacterial degradation of lignin (43, 47), and compounds such as methylamines can be readily produced as degradation products of, for example, choline or betaines (48). Phosphatidylcholines and betaines are abundant in plant tissue as membrane components and osmolytes, respectively (26) and are excreted or leaked into the surrounding sediment through the plant rhizomes and roots. We successfully detected choline (degradation product of phosphatidylcholine), betaines of glycine and proline, and DMSP in seagrass leaves as well as in their rhizomes (Fig. 2 and *SI Appendix*, Table S3). Highest concentrations of choline, betaines, and DMSP were detected in fresh rhizomes from the surface layer of *Posidonia*-covered sediments, and the concentrations decreased in older plant pieces (i.e., recovered from deeper parts of the sediment). This distribution mirrors the distribution of our measured methane production rates, which followed the same trend (Figs. 1*F* and 2).

It is feasible that glycinebetaine, the main plant osmolyte, might act as a direct methane precursor, as methane production from added glycinebetaine was immediate and proceeded linearly over time (*SI Appendix*, Fig. S5*B*). However, betaines as well as cholines might further be fermented to trimethylamine (and other methylamines) (44, 49) that are suitable substrates for methanogens (50). Many fermentative bacteria have a documented capacity to degrade glycinebetaine to form methylamines (51), and our investigated seagrass sediments hosted numerous populations of typical fermenters, including Clostridiales and Vibrionales (Fig. 3), which may be potential candidates for providing methylamines to the methanogens. The abundance of betaines, choline, and DMSP in rhizomes collected from dead seagrass sediments was largely comparable to fresh rhizomes, with the exception of rhizomes collected from the surface layer in which the analyzed compounds were markedly depleted, presumably due to methanogenesis.

**Fig. 5.**
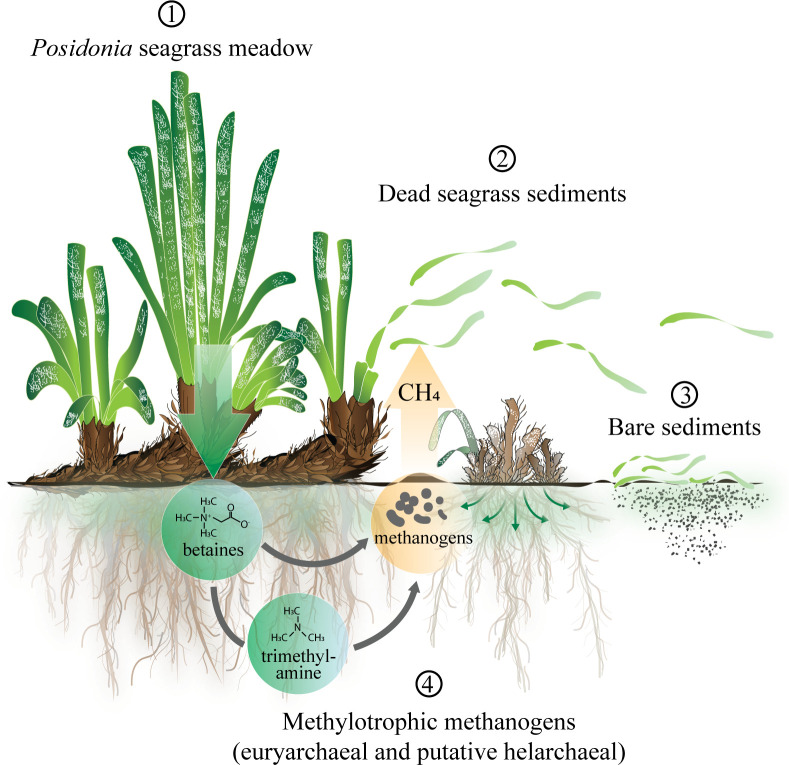
Methane production in vegetated sediments covered by *Posidonia* seagrasses. 1) *Posidonia* seagrasses produce a variety of methylated compounds that can act as methanogenic substrates. For example, betaines are stored in the plant's rhizomes and are released into the surrounding sediment, thereby becoming accessible for degradation. 2) Plant pieces buried in the dead seagrass sediments remain a source of methylated compounds for long periods of time. 3) Detached seagrass leaves that get deposited onto the adjacent dead seagrass or bare sediments may act as an additional source of methylated compounds in this ecosystem. 4) In the sediment, a diverse community of methanogenic archaea produces methane either directly from methylated compounds (e.g., betaines) or their degradation products (e.g., methylamines), mainly in the surface sediment layer.

In addition to rhizomes, seagrass leaves were also found to contain high amounts of all investigated methylated compounds (*SI Appendix*, Table S3). As *Posidonia* plants shed their leaves year-round, we speculate that the resulting leaf debris deposited on the surrounding unvegetated sediment may act as an additional and persistent source of plant-derived methane precursors (Fig. 5).

### Diverse Methylotrophic Methanogens in *P. oceanica* Sediment.

Methylotrophic methanogenesis is generally performed by members of the *Methanosarcinaceae* family (formerly phylum Euryarchaeota, now Halobacteriota). We recovered 16S rRNA gene sequences belonging to this family from our vegetated sediments, albeit at a relatively low abundance (<2% of the archaeal community). Members of the genus *Methanococcoides* were the most abundant euryarchaeal methanogens in all sediments based on 16S rRNA gene abundances. Importantly, we also recovered a full McrA protein sequence from the oxic surface sediment that clustered together with McrA sequences of *Methanococcoides*, with closest phylogenetic affiliation to *M. methylutens*. *M. methylutens* is a marine methanogen originally isolated from a sediment underlying a mat of algae and seagrass debris (52). While it can grow on methylated compounds, it cannot use H_2_ or acetate (52). This fits well to our substrate-addition experiments, where only the addition of methylated compounds resulted in methane production. Other closely related McrA sequences belonged to, for example, a choline-utilizing methanogen isolated from a marine sediment (97.9% amino acid identity to WP_135613124.1) (53). These methanogens have been observed to form syntrophic associations with fermentative bacteria (e.g., *Clostridia* sp., *Pelobacter* sp.), which provide (tri)methylamines from the degradation of glycinebetaine and/or choline (51, 54). However, *Methanococcoides* have also been reported to be capable of using choline and glycinebetaine directly (55, 56) and do not necessarily require a bacterial partner for methane production. In a recent study, *Methanococcoides*, *Methanosarcina*, and *Methanolobus* species were successfully enriched on methylated compounds from *Zostera* seagrass sediments (57). Based on our combined results, we thus propose that members of these cosmopolitan and ubiquitous methylotrophic methanogens were likely responsible for methane production in *P. oceanica* seagrass meadows.

Interestingly, the *P. oceanica*–covered sediments also contained high abundances of other putatively methylotrophic methanogenic archaeal groups—such as *Ca.* Bathyarchaeota (9), Asgard archaea (13), and Thermoplasmata (11). It should be noted that for each of these phyla, only a handful of species is presumably capable of methane metabolism; most Bathyarchaeota, Asgard archaea, and Thermoplasmata are metabolically versatile and are often involved in, for example, lignin and peptide degradation, acetogenesis, and exogenous protein mineralization (58). Additionally, the Mcr proteins from Bathyarchaea and Helarchaea (subgroup of Asgard archaea) belong to the “divergent” Mcr proteins, which typically share very low amino acid sequence homology to the Mcr from classical methanogens. It has thus been proposed that they may not be involved in methane metabolism (59). Instead, the divergent Mcr of *Ca.* Syntrophoarchaeum, for example, has been shown to be involved in butane oxidation (12), and a similar role has been proposed for the divergent bathyarchaeal (59) and helarchaeal Mcr (13).

We retrieved seven McrA protein sequences from vegetated sediments that were affiliated with the clade of “divergent” Mcr sequences (Fig. 4*A*), more specifically, with the McrA sequences from *Ca.* Helarchaea (Fig. 4*C*). Given the lack of evidence for the presence of butane in the investigated sediments, it does not seem immediately obvious that the seagrass-associated Helarchaea should make a living off the oxidation of butane. Instead, given the high potential for methane production from a variety of methylated compounds, we suggest that a possible involvement of this Mcr in methane metabolism should be reexamined. Alternatively, the possibility of Mcr-containing Helarchaea being involved in the conversion of methane precursor molecules as a part of a microbial consortium should be considered. In any case, our combined data show that a diverse community of both traditional and putatively novel methanogenic archaea in these sediments is involved in the production of methane from a variety of plant-derived methylated compounds.

## Conclusions

Our work shows that the high methane fluxes from seagrass sediments are a result of high rates of methylotrophic methanogenesis and a fast advective and plant-mediated transport, combined with an inefficient microbial methane filter in these sediments. High rates of methane production observed in dead seagrass sediments might be explained by a persistent input of various simple as well as larger methylated compounds through the buried plant rhizomes and deposition of fresh leaf debris. As seagrass habitats are declining around the world due to increased eutrophication and physical disturbances of their habitats, these ecosystems lose their ability to sequester carbon dioxide from the atmosphere. However, the capacity of these sediments to produce methane may persist long after the meadow die-off, thus continuing to offset the blue carbon function of these ecosystems in the long run.

## Materials and Methods

### Sampling Site.

Seagrass meadows of *P. oceanica* were studied during three sampling campaigns in October 2018, June 2019, and September 2019 in Fetovaia Bay, Elba, Italy. We visualized the distribution of *P. oceanica* meadows in the Mediterranean Sea according to Telesca et al. (38) using the quantum geographic information system (QGIS; version 3.14). Data for sediment distribution in the Mediterranean Sea was downloaded from the European Marine Observation Data Network (EMODnet; https://www.emodnet.eu, last visit 7/28/2020). In the bay of Fetovaia, *P. oceanica* forms dense meadows in shallow, 7-m–deep water (*SI Appendix*, Fig. S1*A*). Additionally, there are patches of extinct seagrass meadows where the surficial cover of living plants had died off probably more than 25 y ago, leaving behind dead seagrass overlying peat-carrying sediments with highly degraded plant tissue (*SI Appendix*, Fig. S3*A*). We estimate that these meadows died off more than 25 y ago because the dead seagrass patch was already eroded at the time that this site was visited for the first time by M.W. and C.L. We sampled the sediment from an intact seagrass meadow (referred to as “vegetated sediment”) and from an adjacent eroded side that was no longer covered with living seagrass plants (referred to as “dead seagrass sediment”).

### Porewater Sampling and Sediment Coring.

During three sampling campaigns, sediment porewater was retrieved during 13 scuba dives for analysis of oxygen, methane, sulfide, and sulfate. Porewater sampling was performed with a 1-m–long, gas-tight, stainless-steel lance, containing an inner mesh (63 µm) to avoid intake of sediment and plant material. Porewater was extracted from different sediment horizons into polyethylene syringes, and care was taken to avoid formation of gas bubbles and loss of dissolved gases. For determination of dissolved oxygen, the porewater was percolated through an oxygen flow-through cell that was installed between the porewater lance and the syringe (*SI Appendix*, Fig. S8). The oxygen flow-through cell contained an oxygen-sensitive fluorescent dye on the inside, from which dye excitation and emission could be measured contactless from the outside via a glass fiber connected to an underwater oxygen module (Pyroscience, FSO2-SUBPORT). Oxygen concentrations were logged using a diver-operated motorized microsensor system (60). Prior to porewater sampling, the oxygen flow-through cell was calibrated using a two-point calibration procedure by which the flow-through cell was first percolated with aerated seawater (100% oxygen reading) followed by treatment of the seawater with sodium dithionate (0% oxygen reading). Porewater was extracted from the sediment in 10-cm intervals ranging from 0 to 50 cm. For determination of dissolved oxygen, the porewater was extracted with a higher resolution in intervals of 0, 3, 6, 10, 20, 30, 40, and 50 cm. Porewater was sampled in duplicates by extracting two times 20 mL from two adjacent sites to ensure sufficient sample recovery from each depth horizon. Sampling of 20 mL in 10-cm depth intervals results in a sampling halo with a radius of 3.37 cm, indicating that there was no overlap between individual depth horizons.

Immediately after porewater retrieval, the syringes containing the porewater samples were attached to a lift bag and brought to the boat for further processing. The porewater was carefully transferred into a 12-mL exetainer (Labco), fixed in 100 µL saturated mercuric chloride solution, and closed headspace-free for analysis of dissolved methane concentrations. Another 3 mL were fixed in 5% zinc chloride solution for sulfide and sulfate measurements. The remaining 25 mL were sterile filtered and kept at −20 °C for further analysis.

At the same locations, the sediment was sampled with 50-cm–long stainless-steel cores, which were drilled into the sediment by divers (*SI Appendix*, Fig. S1*B*). The cores had sharp edges to cut through the plant material in the sediment. After the cores were removed from the sediment, they were closed with rubber stoppers and kept at 22 °C in a barrel filled with seawater until further processing.

### Whole-Core Incubations for Methane Flux Measurements.

To quantify the methane flux from the sediment into the water column, we incubated whole sediment cores from vegetated sediment (*n* = 11) and from dead seagrass sediment (*n* = 4) in the dark at 22 °C upon arrival of the cores in the laboratory. Vegetated sediment cores were collected within a seagrass meadow, and some of the cores contained an intact plant with leaves, whereas for some cores, the plant leaves were cut off during the coring process. Cores from dead seagrass sediment were collected from a site where the living meadow had died off and did naturally not contain a seagrass plant. Prior to incubation, the cores were filled up with fresh seawater, from which a sample was collected into a serum vial to determine the methane concentration at the start of the incubation (T0). The cores were then closed off gas-tight and incubated at 22 °C for 24 h. At the end of the incubation (after 24 h), another water sample was transferred into a serum vial. All water samples transferred into serum vials were closed off headspace-free with butyl rubber stoppers, crimped, and saturated mercuric chloride solution was added to stop microbial activity. The methane concentration (in ppm) in the sampled water was measured on a cavity ring-down mass spectrometer (G2303, Picarro Inc.) coupled to a closed-loop measurement system. For that, a 20-mL headspace of artificial air was set onto the serum bottles, and dissolved methane was equilibrated with the headspace by vigorous shaking. Methane concentrations (in nmol l^−1^) in the samples were determined using a calibration curve, for which three standards were measured: 1) degassed distilled water (zero calibration), 2) air-saturated distilled water, and 3) air-saturated salt solution (10% sodium chloride).

The methane production rate (in µmol CH_4_ ⋅ l^−1^ ⋅ d^−1^) was calculated based on the change in methane concentration within 24 h. The rates were then converted to an areal flux of methane from the sediment into the water column over a 24-h time period (in µmol CH_4_ ⋅ m^−2^ ⋅ d^−1^) taking into account the core diameter (6.8 cm) and the volume of the overlying water (around 500 mL).

### Sediment Incubations, Stable-Isotope Labeling Experiments, and Isotope Ratio Mass Spectrometry (IRMS).

During three sampling campaigns in October 2018, June 2019, and September 2019, we collected a total of 12 vegetated sediment cores and 10 dead seagrass sediment cores, from which stable-isotope experiments were set up to investigate methane production from different ^13^C-labeled compounds.

The cores typically had a length of 45 cm and were divided into three depths according to their composition, consisting of a sandy top layer with rhizomes and roots from 2- to 10-cm depth (D1), a deeper layer with plant roots between 10 and 30 cm (D2), and a peat layer from 30- to 45-cm depth (D3). The depth horizons were defined based on the sediment appearance (*SI Appendix*, Fig. S1*C*), and their absolute depths thus slightly varied between cores. Thorough care was taken not to expose the sediment core to air, and all core handling was done in a glove bag filled with dinitrogen gas. Sediment material from each depth was homogenized and about 20 g wet weight were added into 120-mL serum bottles, prefilled with sterile, anoxic, helium-degassed seawater. The bottles were closed headspace-free with a butyl rubber stopper and sealed with aluminum crimps (*SI Appendix*, Fig. S1 *D* and *E*). Stable-isotope incubations were set up in separate incubation bottles with either ^13^C-methylamine (purchased from Cambridge Isotope Laboratories), ^13^C_2_-DMA, ^13^C_2_-acetate, ^13^C-methanol, ^13^C-bicarbonate (250 µM) added together with 20 µM hydrogen, or ^13^C_2_-DMS (30 µM), all added at 10-µM final concentration if not indicated differently (all substrates purchased from Sigma Aldrich if not indicated differently). A control experiment was conducted to which 10 mM BES (structural homolog of methyl-CoA; purchased from Sigma Aldrich) had been added together with ^13^C-methylamine in order to inhibit archaeal methanogenesis. A second control sediment incubation was left unamended to measure bulk methane production from substrates readily available in the sediment. Additionally, a set of sediment incubations was amended with 1 mM unlabeled (^12^C-)glycinebetaine (purchased from Sigma Aldrich), from which bulk methane production rates were determined and compared to methane production rates of unamended sediment incubations. To all incubations amended with ^13^C-labeled substrates, nonlabeled methane (100 nM) was added to capture ^13^C-labeled methane production. Both hydrogen and nonlabeled methane were added as dissolved gases that were dissolved in sterile-filtered, anoxic seawater by replacing 1 mL seawater (in a prefilled 12-mL exetainer) with 2 mL respective gas, assuming that both gases will dissolve at their maximum solubility.

The incubations were subsampled at time intervals of 0, 3, 6, 12, and 24 h by removing 6 mL incubation water and simultaneous replacement with sterile, anoxic seawater. The sampled incubation water was transferred into 6-mL exetainers (Labco) containing 100 µL saturated mercuric chloride solution to stop microbial activity and closed headspace-free.

Methane production from ^13^C-labeled compounds was measured by IRMS on a Picarro G2201-i cavity ring-down mass spectrometer coupled to a Liaison interface (A0301, Picarro Inc.) in 3 mL sample acidified with 100 µL 20% phosphoric acid. Methane production from glycinebetaine-amended and unamended sediment incubations were measured via gas chromatography (GC) (*Methane Concentration Measurements via GC*).

### Calculation of Methane Production Rates.

The rates of net methane production were calculated from the slope of linear increase of ^13^C-CH_4_ over ^12^C-CH_4_ over time and normalized for the amount of sediment added per incubation (rates given as pmol per gram sediment wet weight). All rates were inferred from the slope of linear regression across all five time points and are reported only if slopes were significantly different from zero (*P* < 0.05, one-sided Student's *t* test). For all incubations with added ^13^C-labeled methylated compounds, methane concentration increased slightly exponentially over time, which could be due to diffusion limitation of the added tracer in the first hours of the experiment. The methane production rates might thus represent a conservative estimate of the potential rate. For comparison, we also report rates for which T0 values were not included in the rate calculation (*SI Appendix*, Table S2).

### Methane Oxidation Experiments.

Experiments to determine the methane oxidation potential of the sediment community were conducted in October 2018 and September 2019. Sediment incubations were set up as described in *Sediment Incubations, Stable-Isotope Labeling Experiments, and Isotope Ratio Mass Spectrometry (IRMS)*, to which 1 µM ^13^C-labeled CH_4_ (purchased from Cambridge Isotope Laboratories) was added in dissolved form, prepared the same way as described for hydrogen and nonlabeled methane. Anaerobic methane oxidation was investigated in the same sediment horizons as for the other incubation experiments (2 to 10, 10 to 30, and 30 to 45 cm). Anaerobic methane oxidation was investigated in sediment incubations to which no electron acceptor had been added in addition to in situ sulfate. Aerobic methane oxidation was investigated only in the surface sediment layer (2 to 10 cm), for which the incubations were kept at low oxygen concentrations (3.5 ± 2 µM). The oxygen concentration in the incubations was monitored at all sampling time points using oxygen sensor spots (OXSP5, Pyroscience) clued to the inner side of the incubation bottles and read off with optical fibers from the outside. Due to high respiration rates in the sediment, the incubations turned periodically anoxic in between the sampling points. Therefore, they were replenished with new oxygenated water with every sampling. Incubations were subsampled after 0, 3, 6, 12, and 24 h to follow the production of ^13^CO_2_ (as described in *Sediment Incubations, Stable-Isotope Labeling Experiments, and Isotope Ratio Mass Spectrometry (IRMS)*. Production of ^13^CO_2_ from ^13^C-labeled methane was measured by IRMS on a Picarro G2201-i cavity ring-down mass spectrometer coupled to a Liaison interface (A0301, Picarro Inc.). Methane oxidation rates were calculated from the slope of linear increase of ^13^CO_2_ over ^12^CO_2_ over time.

### Methane Concentration Measurements via GC.

Methane concentrations from porewater samples as well as from glycinebetaine-amended and unamended sediment incubations were determined by GC (Agilent Technologies, 7820A GC Systems) equipped with a packed Porapak Q column and a flame ionization detector. Porewater samples that were stored in 12-mL exetainers got a 3-mL helium headspace, from which 0.5 mL were injected into the gas stream of the GC. Incubation samples that were stored in 6-mL exetainers also got a 3-mL headspace, from which 0.5 mL were injected. The removed gas volume was simultaneously replaced by distilled water. Standard calibration was done by injecting different volumes of calibration gas (100 ppm CH_4_ in helium, purchased from Air Liquide).

### Chemical Analysis of Sulfide and Sulfate.

Porewater samples for sulfide and sulfate concentration measurements were fixed in 5% zinc chloride solution directly after sampling. Between 10 and 1,000 µL sulfide samples were diluted with ultrapure water to reach a total sample volume of 2 mL. To each 2-mL sample, 160 µL diamine reagent was added. The sample was immediately closed and left to rest for a minimum of 30 min in the dark at room temperature. The spectrophotometer was zeroed using a cuvette filled with a blank (ultrapure water with the diamine reagent). Samples were briefly shaken, then measured on a spectrophotometer (1-cm path length of cuvette, 670-nm wavelength).

Sulfate concentrations of zinc chloride–treated porewater samples were determined by suppressed ion chromatography (IC) using a Metrohm 761 Compact IC (Metrohm A Supp 5 column) with CO_2_ suppression and online removal of zinc (Metrohm A PPC 1 HC matrix elimination column).

### Extraction and Identification of Methylated Compounds from Plant Tissue.

To determine concentrations of small methylated compounds (choline, betaines, and DMSP) in the plant tissue, we sampled seagrass leaves and rhizomes. For vegetated sediments, seagrass leaves (*n* = 6) were cut off seagrass plants, and seagrass rhizomes were collected from the same sediment depth horizons as used for the incubation experiments, if present (two to five rhizomes per depth). Rhizomes from the uppermost depth horizon were intact but partly degraded in the deeper depth horizons. From dead seagrass sediment cores that did not have a surficial cover of living seagrass plants, only the rhizomes from the different depth horizons were collected (two rhizomes per depth). Plant tissue samples were cleaned from sediment by rinsing them with MilliQ and kept at −20 °C until extraction. Directly before extraction, the plant pieces were freeze dried and ground to a fine powder. About 50 mg powder per sample were extracted with 1 mL solvent mixture (61). A total of 50 µL each extract was dried in a vacuum rotation device and after resuspended in 50 µL water. Subsequently, 1 µL sample and 1 µL standard mixtures (1 to 100 µM) were spotted on a target for matrix-assisted laser desorption ionization mass-spectrometry. After drying, dihydroxybenzoic acid matrix was used to coat the sample and analyzed using an AP-SMALDI10 source and an Orbitrap QExactive mass-spectrometer as described previously (62). Identity and concentrations of methylated compounds were determined by comparing measured *m/z* values to exact masses of choline, glycinebetaine, and DMSP and by comparing ion intensity to measured standard mixtures. DMSP was not contained in the standard mixture and concentrations were estimated from measured ion intensities of glycinebetaine standards.

### Nucleic Acid Extraction and Metagenome Sequencing.

DNA was extracted from sediment samples taken in October 2018, as well as in June and September 2019 from the same sediment cores and sampling depths as used for stable-isotope incubations. Sediment samples were frozen and kept at −80 °C until DNA extraction. DNA was extracted from 2 g sediment with the DNeasy PowerSoil Kit (Qiagen) according to the manufacturer's instructions and quantified with the Qubit double-stranded DNA HS Assay Kit on a Qubit 2.0 fluorometer (Invitrogen). Library preparation and sequencing were performed at the Max Planck Genome Center Cologne, Germany (https://mpgc.mpipz.mpg.de/home/). A total of 10 ng genomic DNA was used for library preparation with NEBNext Ultra II FS DNA Library Prep Kit for Illumina (New England Biolabs). Library preparation included eight cycles of PCR amplification. Quality and quantity were assessed at all steps via capillary electrophoresis (TapeStation, Agilent Technologies) and fluorometry (Qubit, Thermo Fisher Scientific). Libraries were sequenced on HiSeq2500 system (Illumina) with 2 × 250 base pair (bp) paired-end reads (*SI Appendix*, Table S1).

Additionally, two samples (C3_D1 and C3_D3) were sequenced using PacBio Sequel II. DNA was quality assessed by capillary electrophoresis (FEMTOpulse, Agilent), and barcoded low-input PacBio libraries were prepared as recommended by PacBio with the SMRTbell Express Template Prep Kit 2.0 (Pacific Biosciences) without an additional fragmentation. Complexes consisting of library fragments/polymerase/primer were built with Sequel II Binding Kit 2.0 and sequenced with Sequel II Sequencing chemistry 2.0 on a single molecule real-time (SMRT) cell (8 M zero-mode waveguides) for 30 h with a final library concentration of 70 pmol on plate in high-fidelity (HiFi) mode. The total output was 290.86 gigabase continuous long read data. HiFi data were extracted from the two pooled libraries yielding 7.7 and 1.8 gigabases, respectively.

### Microbial Community Profiling and 16S rRNA Gene Phylogeny.

Microbial community composition based on 16S rRNA gene sequences in raw metagenomes was performed using phyloFlash (version 3.3b2) (63) and the SILVA database (release 138) (64). Prior to calculation of relative abundances, reads phylogenetically assigned to mitochondria, plastids, and Eukarya were removed. Additionally, full-length 16S rRNA gene sequences taxonomically assigned to Bathyarchaeia were assembled from metagenomic 16S rRNA gene sequences using SPAdes assembler version 3.11.1 (65) as implemented in phyloFlash. A neighbor-joining tree using Jukes–Cantor substitution model with 1,000 bootstrap iterations was calculated in ARB (version 6.1) (66) from 98 representative 16S rRNA gene sequences (longer than 900 bp) of uncultivated Bathyarchaeia (37, 67) and our metagenome-recovered 16S rRNA gene sequences that classified as Bathyarchaeia. 16S rRNA gene sequences of 12 cultivated Thaumarchaeota species were used as an outgroup. Sequences were aligned using SINA web aligner (version 1.2.11) (68).

### Identification and Phylogeny of Metagenomic McrA Protein Sequences.

Adapter and quality trimming of raw metagenomic Illumina reads was performed using Trimmomatic 0.32 (69) (parameters: LEADING:3 TRAILING:3 SLIDINGWINDOW:4:10 MINLEN:200), and each metagenomic Illumina dataset obtained from 2019 was assembled individually using metaSPAdes 3.13.0 (70) and standard parameters. Furthermore, Illumina metagenomes from 2018 (C1 to C4, depth D3) were combined and coassembled as described for individual metagenomic datasets. The PacBio CCS (circular consensus sequence) reads were generated and demultiplexed by PacBio SMRT Link version 8 and default settings. Afterward, the bam2fastq tool, shipped with SMRT Link, was used to convert resulting xml files into fastq format.

For identification of *mcrA* gene sequences in contigs of Illumina assemblies (minimum length > 750 bp for coassembly of 2018 metagenomes, no minimum length for other assemblies) and PacBio CCS, protein-coding sequences were predicted using prodigal 2.6.3 (71) in metagenomic mode, and amino acid sequences were subsequently searched for McrA sequences using hmmscan (of the HMMer package) (72) and McrA HMM models obtained from PFAM (PF02249.17, PF02745.15) (73) using trusted cut-offs (–cut_tc).

McrA protein sequences from the vegetated sediment metagenomes were added to the alignment published by Boyd et al. (74) comprising classical and divergent McrA protein sequences using the MAFFT alignment program (version 7). A Maximum likelihood tree from 214 McrA protein sequences was calculated with IQ-TREE multicore version 1.6.11 (75) using model LG+F+I+G4 and 1,000 bootstrap iterations. The constructed McrA phylogenetic tree was modified with itol webtool (https://itol.embl.de) (76). Amino acid sequence identities were determined with Blast (https://blast.ncbi.nlm.nih.gov/Blast.cgi).

## Supplementary Material

Supplementary File

Supplementary File

Supplementary File

Supplementary File

Supplementary File

Supplementary File

Supplementary File

Supplementary File

## Data Availability

The sequence data generated in this study are deposited in the NCBI database (https://www.ncbi.nlm.nih.gov/) under BioProject number PRJNA788475. Supplementary files are attached to this manuscript that contain McrA sequences recovered from sediment metagenomes (Dataset S1), McrA amino acid sequences (Dataset S2), 16S rRNA gene sequences classified as *Ca*. Bathyarchaeota (Dataset S3), assembled archaeal 16S rRNA gene sequences (Dataset S4) and corresponding classification (Dataset S5), raw output from Phyloflash as presented in Fig. 3 (Dataset S6), as well as accession numbers used as base for *SI Appendix*, Fig. S6 (Dataset S7). Additionally, these datasets are publicly accessible from the MPG Data Repository (EDMOND; https://edmond.mpdl.mpg.de/imeji/collection/YYxLiBnJfvo3oVV) under the same file names. Publicly available sequences used for phylogenetic tree construction (Fig. 4 and *SI Appendix*, Fig, S6, Dataset S7) can be found under their respective accession numbers at NCBI. All other study data are included in the article and/or supporting information.
