# Supplementary material for: Diverse methylotrophic methanogenic archaea cause high methane emissions from seagrass meadows
Source: Proc Natl Acad Sci U S A. 2022 Feb 14;119(9):e2106628119. doi: 10.1073/pnas.2106628119 (PMC8892325; doi:10.1073/pnas.2106628119)
Supplement: Supplementary File [file pnas.2106628119.sapp.pdf]

Diverse methylotrophic methanogenic archaea cause high methane emissions from seagrass meadows

Sina Schorn, Soeren Ahmerkamp, Emma Bullock, Miriam Weber, Christian Lott, Manuel Liebeke, Gaute Lavik, Marcel M. M. Kuypers, Jon S. Graf, and Jana Milucka

SUPPLEMENTARY MATERIAL

I. Datasets

Dataset S1: Information on recovered mcrA sequences from sediment metagenomes (excel file)

Dataset S2: McrA protein sequences recovered from Illumina and PacBio sequencing (text file)

Dataset S3: 16S rRNA gene sequences classified as *Ca.* Bathyarchaeota recovered from sediment metagenomes (fasta file)

Dataset S4: Assembled archaeal 16S rRNA sequences (fasta file)

Dataset S5: Classification of assembled 16S rRNA gene sequences belonging to supplementary file 4 (classification file)

Dataset S6: Raw output from PhyloFlash used as base for Figure 3 (excel file)

Dataset S7: Accession numbers used as base for Figure S6 (excel file)

II. Supplementary Text

III. Supplementary Methods

IV. Supplementary Figures

V. Supplementary Tables

SUPPLEMENTARY TEXT

Text S1. Characteristics of the sediment underlying a *Posidonia oceanica* meadow and the implications for methane fluxes

The investigated *Posidonia* meadow off the coast of Fetovaia Bay, Elba, grows on sandy sediments; in fact, the Mediterranean coastline largely consists of sandy sediments (Fig. S2A). Grain sizes from the sediment underneath a living seagrass meadow ranged from < 50 µm to 800 µm with a median size of 177 µm (Fig. S2 B and C). Typically, sand classifies as grain sizes from 63 µm to 2 mm, whereas mud is defined to have particle sizes below 63 µm (1). Therefore, the majority of grains can be classified as fine sand. Additionally we investigated the sediment porosity of vegetated and dead seagrass sediments. In the surface layer of vegetated sediment (0-6 cm) we determined a constant porosity of 0.4±0.04 among four replicate measurements, typical of sandy sediments (2). In the deeper sediment layers (6-40 cm) the average porosity was 0.47±0.09 and showed a much higher variability, which can be assigned to the heterogeneity of the sediment that is interspersed by plant tissue. The porosity of the dead seagrass sediment (measured on three cores) was similar to that of the vegetated sediment (Fig. S2D).

In sandy sediments porewater advection leads to a more rapid water exchange as compared to muddy sediments (2, 3). Our porewater gas profiles of vegetated sediments did not show subsurface methane accumulation typical for muddy sediments. Even though the porewater methane concentrations were supersaturated with respect to atmospheric equilibrium (*ca.* 2 nM), the concentrations were only in the nanomolar range (29-132 nM) and stayed constant throughout the investigated upper 50 cm of sediment, implying rapid exchange between the sediment porewater and the water column. A slight decrease in methane concentrations was only detected at the sediment surface, which might indicate loss of methane from the sediment to the overlying water column.

Overall, it is thus expected that methane will rapidly exchange between the sediment, water column and the atmosphere in these vegetated sediments due to the shallow water depths of the seagrass beds and their growth in sandy sediments that support advective transport.

Text S2. Methane emissions and carbon offset of *Posidonia oceanica* meadows

Upscaled, the total methane emissions from *P. oceanica* meadows into the water column in the Mediterranean Sea range from 0.0003 to 0.033 Tg CH_4 yr^{-1}. Due to the shallow depth of the seagrass beds (7 m) the methane released into the mixed water column is not expected to undergo any significant oxidation before exchanging with the atmosphere. Thus, assuming that the methane flux to the water column equals that to the atmosphere, then *P. oceanica*-associated emissions from the Mediterranean Sea would represent between 0.3-1.2 % of the global methane emissions from seagrass ecosystems (0.1-2.7 Tg yr^{-1}) (1, 4, 5). The emissions of methane from the vegetated sediment offset the effect of CO_2 uptake by the plant - both in terms of carbon storage as well as the global warming effect. In seasons of high productivity (early summer) *P. oceanica* showed an average net ecosystem metabolism (i.e. carbon storage described as its production (photosynthesis) minus its consumption (respiration)) of 77 mmol CO_2 m^{-2} d^{-1} (6). Our measurements show median methane emissions of 106 µmol CH_4 m^{-2} d^{-1} of the vegetated sediments. This means that only a negligible fraction of the fixed carbon is lost from the ecosystem as methane (*ca.* 0.001%). However, due to the higher warming potential of methane over carbon dioxide (28 to 36 times higher; (7)), between 4 and 5% of the blue carbon function of this plant might be offset by its methane-producing capacity.

Text S3. Methane production in dead seagrass sediments

In the bay of Fetovaia, there are sites where the living seagrass plants have died off in the past due to physical disturbance (Fig. S3A). The sediment under these eroded seagrass beds still contains the peat

substrate consisting of dead plant material but is no longer interspersed by the living plant tissue. Based on the chemical porewater profiles, there was a profound difference between sediments covered with living and dead seagrass, respectively (Fig. 1 C, D, and E and Fig. S3 B and C). Most apparent was the high accumulation of methane and free sulfide in the porewater of dead seagrass sediments. Clearly, the living plant alters the sediment biogeochemistry, and after its disappearance certain functions of the plant are no longer maintained. One of these functions is the ventilation of the sediment by periodic oxygen intrusions through the plant tissue (8), which may control the accumulation of free sulfide. Downward oxygen transport by the plant can occur passively through gas diffusion driven by differences in partial pressures between leaves and rhizomes or through mass flow inside the plant driven by a 'pumping' effect induced by the plant movement through the action of waves (9). The absence of the living plant as a conduit for gas exchange between the sediment and the water column might also explain the higher methane accumulation in dead seagrass sediments.

In contrast to the porewater profiles, the potential rates of methane production in the dead seagrass sediments were largely similar to those in the vegetated ones (Fig. 2 and Fig. S4). Only in the deepest sediment depth, below 30 cm, the rates of methane production were notably lower. This implies that significant capacity for methane production in seagrass sediments may remain even long after the disappearance of the living plant.

Text S4. Calculation of tracer-derived methane production rates

Even though the tracer-derived rates measured in the vegetated sediment appear lower than in the dead seagrass sediment (1,017 pmol g_{sed}^{-1} d^{-1} vs. 2,897 pmol g_{sed}^{-1} d^{-1}, respectively), the total 'real' rates of methane production might be in fact higher in the vegetated sediment where the background concentration of the non-labeled substrate is higher, i.e. the labeling percentage of the substrate is lower. This is corroborated by the fact that in unamended incubations (i.e. with residual *in situ* substrate concentrations) methane production rates in the dead seagrass sediment were lower than in the vegetated ones.

SUPPLEMENTARY METHODS

Determination of sediment porosity

We determined the sediment porosity from four vegetated sediment cores and from three dead seagrass sediment cores. Subcores of 3 cm^3 were sampled in intervals of 7 to 10 cm over a depth of up to 50 cm. 2 ml of sediment was added into a cylinder, weighed and dried at 60°C. The porewater volume was then determined by calculating the difference of sediment wet weight and dry weight divided by the seawater density. Subsequently, the sediment porosity (cm^3 porewater cm^{-3} wet sediment) was determined by dividing porewater volume by the total volume of the wet sediment.

From a subsample we determined the grain size of the sediment from a total of 500 sand grains sampled from the upper 6 cm of the vegetated sediment. Sand grains were equally and randomly distributed in a petri dish and imaged using a Leica DMI 6000B inverted microscope. The diameter (in pixels) was then measured manually using an in-house developed matlab script (Mathworks R2017b) and converted into µm units based on a reference scale measurement.

SUPPLEMENTARY FIGURES

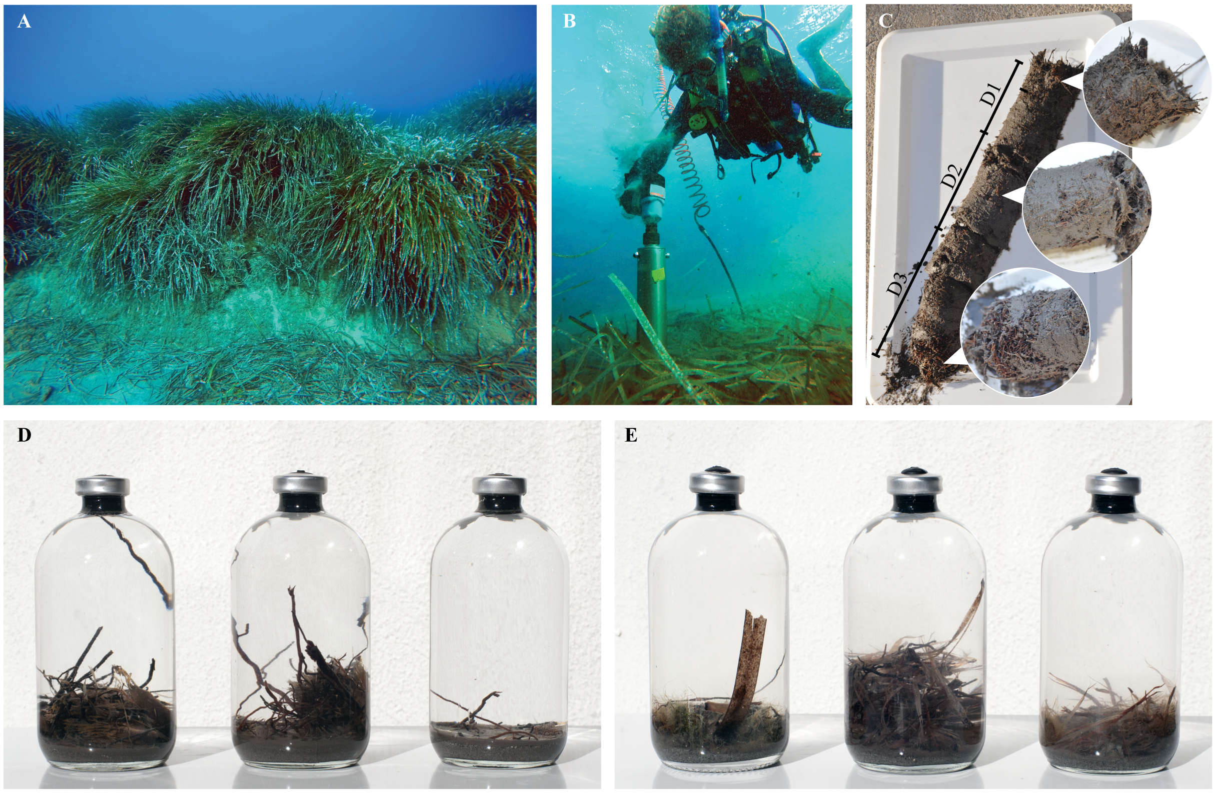

Figure S1. Sampling site and sediment work. (A) Seagrass meadow of *Posidonia oceanica* located in 7 m deep water in the bay of Fetovaia, Elba, Italy. (B) Sediment cores were retrieved with an underwater operated pressure drill. (C) Composition of a 50 cm long sediment core sampled from underneath a seagrass meadow. (D, E) Incubation bottles with approximately 20 g of sediment and peat material from vegetated and dead seagrass sediment. (D) Incubation bottles with sediment from a vegetated sediment core from different depth horizons of 2-10 cm, 10-30 cm, and 30-45 cm (from left to right). (E) Corresponding incubation bottles from dead seagrass sediment cores from the same depth horizons as shown in D. The sediment material was much more degraded in these dead seagrass sediment cores but roots and rhizomes were still discernible in the peated material.

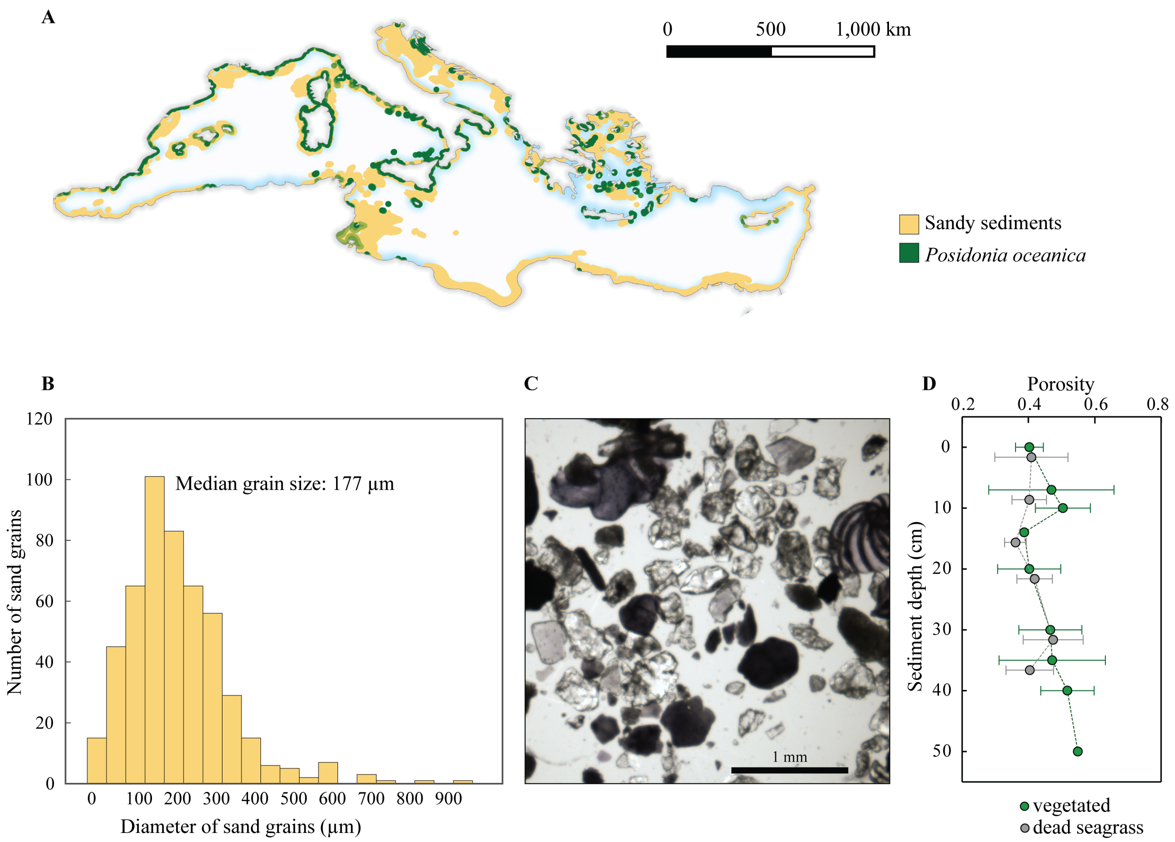

Figure S2. Properties of vegetated and dead seagrass sediments. (A) Distribution of *Posidonia oceanica* (green, after (10)) along with sandy sediments (yellow, source: Emodnet) in the Mediterranean Sea. (B) Grain size distribution from the upper 6 cm of sediment underneath a seagrass meadow, as visualized in (C). (D) Porosity of vegetated and dead seagrass sediments.

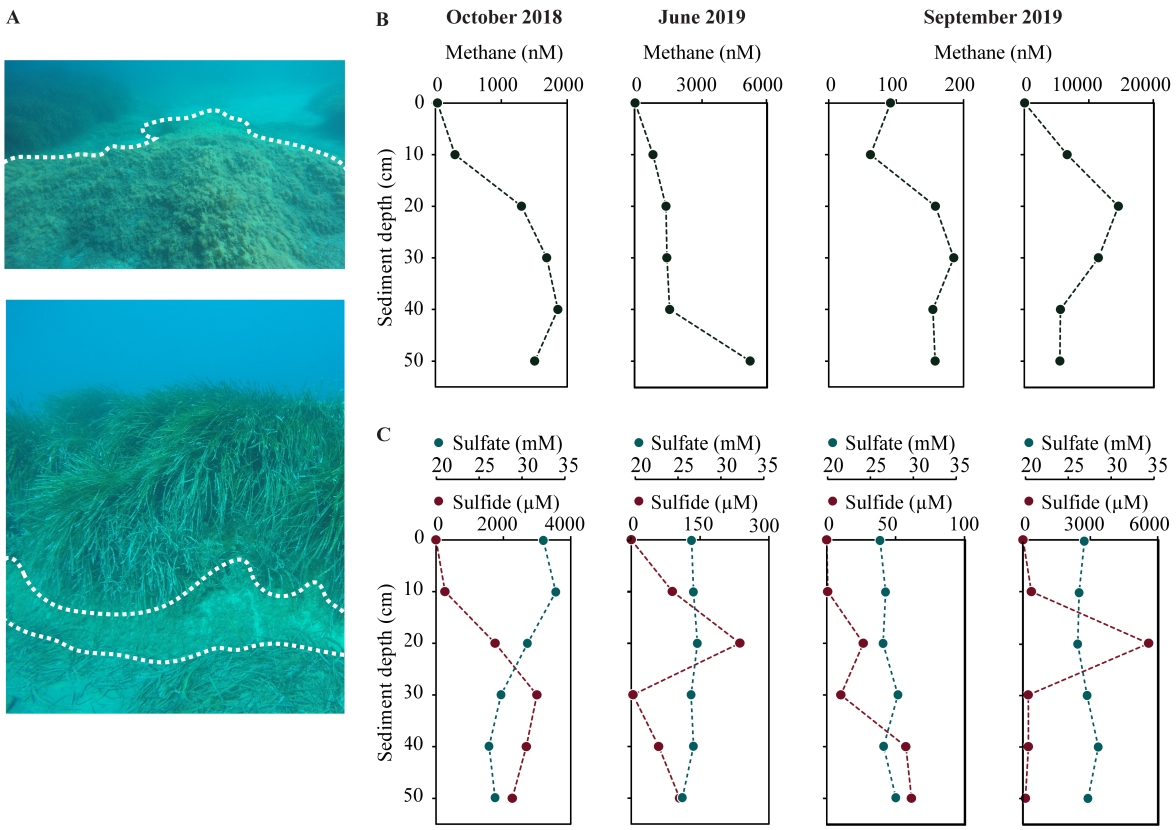

Figure S3. Porewater profiles of dead seagrass sediment. (A) Dead seagrass sediment in the bay of Fetovaia which was formerly colonized by *P. oceanica* seagrasses (top image) as well as peat deposition underneath a living seagrass meadow that persists after seagrass die-off (bottom image). Respective areas are outlined by white dashed lines. (B) Methane porewater concentrations measured in October 2018, June 2019, and September 2019. (C) Corresponding porewater concentrations of sulfide (red) and sulfate (blue).

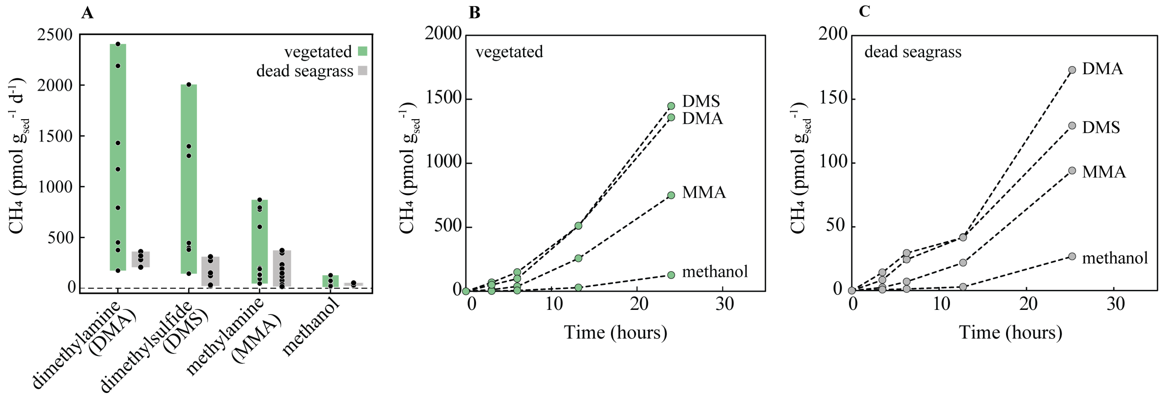

Figure S4. Methane production from different methylated, non-competitive substrates in incubations of vegetated and dead seagrass sediments. (A) ^{13}C-labeled substrates were added to sediment incubations to test for methane production from dimethylamine (DMA), dimethylsulfide (DMS), methylamine (MMA), and methanol. Exemplary representation of methane production over time for the different methylated substrates in vegetated (B) and dead seagrass sediments (C). Methane production rates from methylated compounds presented here were analyzed only in the deepest depth horizon (30-45 cm). All ^{13}C-labeled substrates were added at a final concentration of 10 µM, except for DMS (30 µM). Abbreviations are: DMA, dimethylamine; DMS, dimethylsulfide; MMA, monomethylamine.

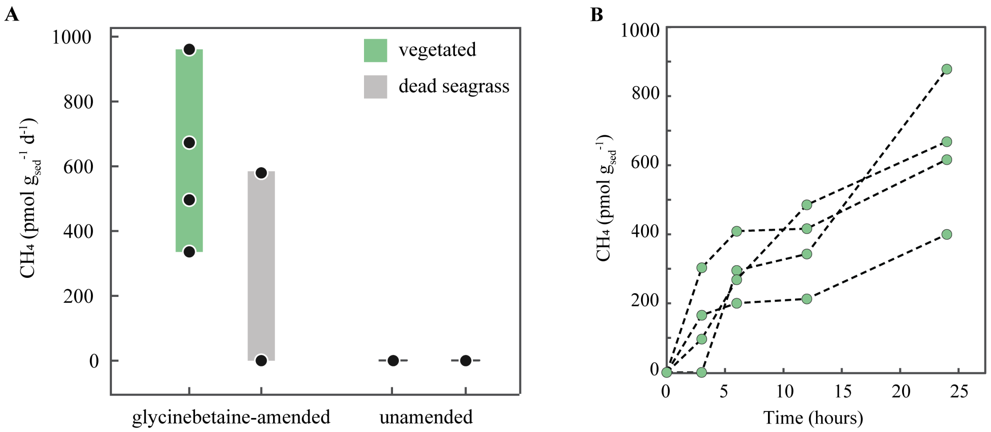

Figure S5. Methane production in ([12]C-)glycinebetaine-amended and unamended incubations of vegetated and dead seagrass sediments. (A) Methane production rates in glycinebetaine-amended and unamended incubations of vegetated and dead seagrass sediment incubations. (B) Methane production in glycinebetaine-amended vegetated sediment incubations over a 24-hour time period. Glycinebetaine was added at a final concentration of 1 mM. Methane production was analyzed in the deepest depth horizon (30-45 cm) over a time course of 24 hours.

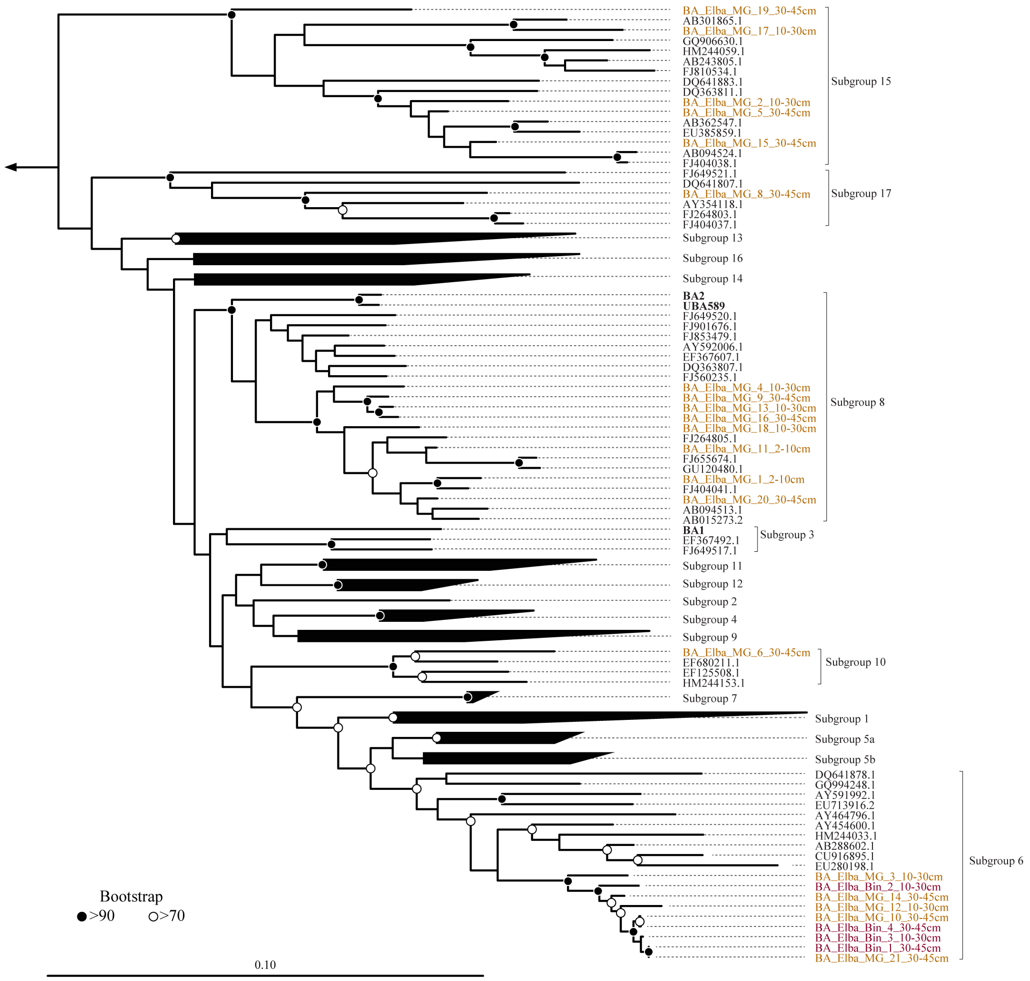

Figure S6. Placement of metagenome-recovered 16S rRNA genes classifying as *Ca.* Bathyarchaeota. The neighbour joining tree (1,000 bootstraps iterations) with 98 representative 16S rRNA gene sequences of uncultured Bathyarchaeia (formerly MCG) (11, 12) showing the placement of metagenome-recovered Bathyarchaeia 16S rRNA gene sequences. We assembled twenty 16S rRNA gene sequences from metagenomic reads (orange) and four 16S rRNA gene sequences from metagenome assembled bins (red) that affiliated with 5 subgroups of the phylum *Ca.* Bathyarchaeota, comprising subgroups 6, 8, 10, 15, and 17. 16S rRNA gene sequences of 12 cultivated Thaumarchaeota species were used as an outgroup. Bootstrap support is shown with white (>70%) and black (>90%) circles. Scale bar indicates substitutions per site.

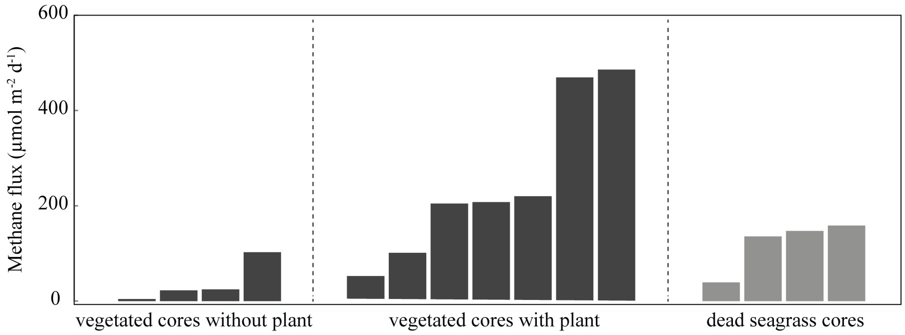

Figure S7. Methane fluxes from whole core incubations of vegetated and dead seagrass sediments. Some cores sampled from vegetated sediment (from within the seagrass meadow) contained a plant with leaves (referred to as "vegetated cores with plant") whereas for some cores the plant leaves were cut off during the coring and were therefore not contained in the core (referred to as "vegetated cores without plant"). Cores sampled from dead seagrass sediment did naturally never contain a plant as the surficial cover of plants had died off. Fluxes of vegetated cores with plants were significantly different from those determined from vegetated cores without plants, but not from dead seagrass sediment cores (one-way ANOVA, $p = 0.037$ and 0.109, respectively).

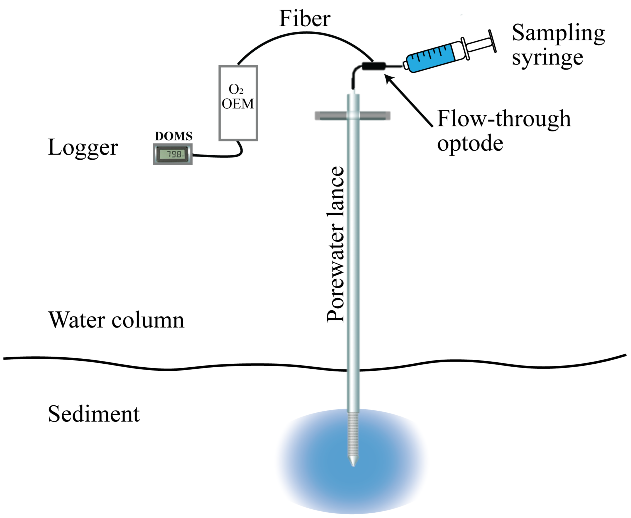

Figure S8. Porewater was sampled using scuba diver-operated stainless steel push-point lances. In order to monitor oxygen concentrations instantly, an oxygen flow-through cell (Pyroscience) was connected between porewater lance and the polyethylene syringe. The cell was connected via a glass-fiber to a read-out module and underwater logger (DOMS) and porewater oxygen concentrations were determined at the sediment-water interface (0 cm) and at depths of 3, 6, 10, 20, 30, 40, and 50 cm. Additionally, porewater samples were collected at 0, 10, 20, 30, 40 and 50 cm for analysis of dissolved gases and nutrients. At each depth horizon, 20 ml of porewater were carefully extracted via a PE syringe that was connected to the lance using gas-tight fluorocarbon tubing (Viton). After sampling, the syringes from each depth horizon were immediately transferred to the water surface for further processing. The combination of 10 cm depth horizons and 20 ml extraction volume results in a sampling halo with a radius of 3.37 cm, indicating that there was no overlap between individual depth horizons.

SUPPLEMENTARY TABLES

Table S1. Summary of raw metagenomic sequences used in this study.

Library name	Sequencing technology	No. of reads (paired-end)	Total sequenced (Gbp)	Sample origin and date
4521_A	HiSeq2500 (2×250 bp)	21,280,150	10.6	Fetovaia Bay, seagrass bed core C6, 2-10 cm, September 2019
4521_B	HiSeq2500 (2×250 bp)	21,920,511	11.0	Fetovaia Bay, seagrass bed core C6, 10-30 cm, September 2019
4521_C	HiSeq2500 (2×250 bp)	21,254,640	10.6	Fetovaia Bay, seagrass bed core C6, 30-45 cm, September 2019
4521_D	HiSeq2500 (2×250 bp)	17,439,745	8.7	Fetovaia Bay, seagrass bed core C3, 2-10 cm, September 2019
4521_E	HiSeq2500 (2×250 bp)	18,798,846	9.4	Fetovaia Bay, seagrass bed core C3, 10-30 cm, September 2019
4521_F	HiSeq2500 (2×250 bp)	21,067,396	10.5	Fetovaia Bay, seagrass bed core C3, 30-45 cm, September 2019
4521_G	HiSeq2500 (2×250 bp)	18,188,497	9.1	Fetovaia Bay, seagrass bed core C2, 2-10 cm, June 2019
4521_H	HiSeq2500 (2×250 bp)	20,236,526	10.1	Fetovaia Bay, seagrass bed core C2, 10-30 cm, June 2019
4521_I	HiSeq2500 (2×250 bp)	21,658,672	10.8	Fetovaia Bay, seagrass bed core C2, 30-45 cm, June 2019
4149_A	HiSeq2500 (2×250 bp)	16,018,078	8.0	Fetovaia Bay, seagrass bed core C1, 30-45 cm, October 2018
4149_B	HiSeq2500 (2×250 bp)	20,604,031	10.3	Fetovaia Bay, seagrass bed core C2, 30-45 cm, October 2018
4149_C	HiSeq2500 (2×250 bp)	15,815,141	7.9	Fetovaia Bay, dead seagrass sediment C3, 30-45 cm, October 2018
4149_D	HiSeq2500 (2×250 bp)	15,801,501	7.9	Fetovaia Bay, dead seagrass sediment C4, 30-45 cm, October 2018
4575_A	PacBio Sequel II (CCS reads)	1,376,013	-	Fetovaia Bay, seagrass bed core C3, 2-10 cm, September 2019
4575_B	PacBio Sequel II (CCS reads)	284,325	-	Fetovaia Bay, seagrass bed core C3, 30-45 cm, September 2019

Table S2. Methane production rates from methylamine-amended incubations (of four incubations per depth). Rates were calculated from all five time points sampled over 24 hours (referred to as "all time points") and from only the last four time points (referred to as "T2 - T5"), omitting T0. Rate calculations from only the last four time points gave a better linear regression (R^2) than including all five time points into the calculation. The average rate per depth was 10 to 12% higher by only taking the linear regression of the four last time points, in comparison to taking all five time points. Significance (p value) was tested using one-tailed student's t-Test.

	All time points (1)			T2 - T5 (2)		
Sediment depth	Rate ($nM\ d^{-1}$)	R^2 (p value)	Average per depth ($nM\ d^{-1}$)	Rate ($nM\ d^{-1}$)	R^2 (p value)	Average per depth ($nM\ d^{-1}$)
D1 2-10 cm	883	0.94 (0.003)		975	0.96 (0.003)	
	1252	0.93 (0.004)		1407	0.96 (0.004)	
	627	0.94 (0.003)		700	0.97 (0.002)	
	1305	0.98 (0.001)	1017	1399	0.99 (0.0004)	1120
D2 10-30cm	106	0.94 (0.003)		117	0.96 (0.003)	
	761	0.95 (0.003)		845	0.97 (0.002)	
	718	0.92 (0.005)		809	0.96 (0.004)	
	229	0.95 (0.003)	453	255	0.97 (0.002)	506
D3 30-45 cm	46	0.94 (0.003)		51	0.96 (0.003)	
	92	0.88 (0.009)		105	0.92 (0.008)	
	126	0.94 (0.003)		141	0.97 (0.002)	
	775	0.94 (0.003)	260	867	0.97 (0.002)	291

(1) The rate of methane production was calculated from all five time points sampled over 24 hours.

(2) For comparison, the rate of methane production was calculated from only the last four time points, omitting T0, assuming that the added tracer first needed to equilibrate with the unlabeled methylamine in the incubation bottle.

Table S3. Concentration of betaines, choline, and dimethylsulfoniopropionate (DMSP) in seagrass rhizomes and leaves collected from vegetated and dead seagrass sediments.

Compound	Plant tissue, sampling depth	Vegetated sediments (average concentration in µmol $g_{(dry\ weight)}^{-1}$); (n)	Dead seagrass sediments (average concentration in µmol $g_{(dry\ weight)}^{-1}$); (n)
Betaines	Leaves	0.3 (n = 6)	no data
	Rhizomes, 2-10 cm	0.8 (n = 5)	0.0 (n = 2)
	Rhizomes, 10-30 cm	0.2 (n = 3)	0.8 (n = 2)
	Rhizomes, 30-45 cm	0.1 (n = 2)	0.9 (n = 2)
Choline	Leaves	10.3 (n = 6)	no data
	Rhizomes, 2-10 cm	8.2 (n = 5)	0.2 (n = 2)
	Rhizomes, 10-30 cm	2.3 (n = 3)	10.9 (n = 2)
	Rhizomes, 30-45 cm	0.6 (n = 2)	0.8 (n = 2)
DMSP	Leaves	179.3 (n = 6)	no data
	Rhizomes, 2-10 cm	93.2 (n = 5)	7.8 (n = 2)
	Rhizomes, 10-30 cm	0.9 (n = 3)	191.0 (n = 2)
	Rhizomes, 30-45 cm	1.6 (n = 2)	1.5 (n = 2)

Bibliography

1. Folk RL. Petrology of sedimentary rocks; Syllabus. 1980.

2. Ahmerkamp S, Winter C, Krämer K, Beer D de, Janssen F, Friedrich J, et al. Regulation of benthic oxygen fluxes in permeable sediments of the coastal ocean. Limnol Oceanogr. 2017 Sep; 62(5):1935-54.

3. Huettel M, Berg P, Kostka JE. Benthic exchange and biogeochemical cycling in permeable sediments. Ann Rev Mar Sci. 2014; 6:23-51.

4. Garcias-Bonet N, Duarte CM. Methane production by seagrass ecosystems in the Red Sea. Front Mar Sci. 2017 Nov 7; 4.

5. Al-Haj AN, Fulweiler RW. A synthesis of methane emissions from shallow vegetated coastal ecosystems. Glob Chang Biol. 2020 Mar 16; 26(5):2988-3005.

6. Koopmans D, Holtappels M, Chennu A, Weber M, de Beer D. The response of seagrass (*Posidonia oceanica*) meadow metabolism to CO_2 levels and hydrodynamic exchange determined with aquatic eddy covariance. Biogeosciences Discussions. 2018 Apr 27; 1-23.

7. IPCC. Climate Change 2014: Mitigation of Climate Change, Intergovernmental Panel on Climate Change, New York, USA. 2014.

8. Oremland RS, Taylor BF. Diurnal fluctuations of O_2, N_2, and CH_4 in the rhizosphere of *Thalassia testudinum* 1. Limnol Oceanogr. 1977 May; 22(3):566-70.

9. Borum J, Sand-Jensen K, Binzer T, Pedersen O, Greve TM. Oxygen movement in seagrasses. Seagrasses: biology, ecology and conservation. Dordrecht: Springer Netherlands; 2006. p. 255-70.

10. Telesca L, Belluscio A, Criscoli A, Ardizzone G, Apostolaki ET, Fraschetti S, et al. Seagrass meadows (*Posidonia oceanica*) distribution and trajectories of change. Sci Rep. 2015 Jul 28; 5:12505.

11. Kubo K, Lloyd KG, F Biddle J, Amann R, Teske A, Knittel K. Archaea of the Miscellaneous Crenarchaeotal Group are abundant, diverse and widespread in marine sediments. ISME J. 2012 Oct; 6(10):1949-65.

12. Meng J, Xu J, Qin D, He Y, Xiao X, Wang F. Genetic and functional properties of uncultivated MCG archaea assessed by metagenome and gene expression analyses. ISME J. 2014 Mar; 8(3):650-9.
