# Supplementary material for: Diverse methylotrophic methanogenic archaea cause high methane emissions from seagrass meadows
Source: Proc Natl Acad Sci U S A. 2022 Feb 14;119(9):e2106628119. doi: 10.1073/pnas.2106628119 (PMC8892325; doi:10.1073/pnas.2106628119)
Supplement: Supplementary File [file pnas.2106628119.sd03.rtf]

>BA_Elba_MG_1_2-10cm
ATCCGGTTGATCCTGCCGGACCTTACTGCTATCGGGGTGGGACTAAGACATGCGAGTCGAGCGTCTCTAGCTATGTTGAGACGCGGCAGACGGCTCAGTAACACGTGGCTAACCTACCCTCAGGACGAGTACAAACCCGGGAAACTGGGGCTAATACTCGATAGGTGAGAGTATCTGGAATGATTTTTCACTCAAAGGGCTTTGGATACATGCTTCCAACGTCGCCTGGGGATGGGGCCGCGTCCGATCAGGTTGTTGGTGAGGTAACGGCTCACCAAGCCTATAACCGGTACGGGCCGTGAGAGCGGTAGCCCGGAGATGGGTACTGAGACAAGGACCCAGGCCCTACGGGGCGCAGCAGTCGCGAAAACTCCGCAATGCACGAAAGTGTGACGGGGCTACCCCGAGTGCCATCCGCTGAGGGTGGCTTTTCTTTGGTATAATTAGCCATTGGAATAAGGAGAGGGCAAGACTGGTGTCAGCCGCCGCGGTAATACCAGCTCTCCGAGTGGTAGGGATGATTATTGGGCTTAAAGCGTCCGTAGCCGGCTTAGCAAGTTTCCTGTTAAACTCAGCGACTCAATCGTTGACCCGCGGGAAATACTACTAGGCTAGGAGGCGGGAGAGGTCGACGGTACTTCTGGGGTAGGGGTGAAATCCTATAATCCCGGGAGGACCACCAGTGGCGAAGGCTGTCGACTAGAACGCGCTCGACGGTGAGGGACGAAAGCTGGGGGAGCGAACCGGATTAGATACCCGGGTAGTCCCAGCCGTAAACGATGCGAGCTAGGTGTTGGGATGGCTACGTGCCATTTCAGTGCCGCAGGGAAGCCATTAAGCTCGCCGCCTGGGGAGTACGGCCGCAAGGCTGAAACTTAAAGGAATTGGCGGGGGAGCACCACAAGGCGTGAAGCTTGCGGTTCAATTGGAGTCAACGCCGGGAACCTTACCGGGGGCGACAGCAGAATGAAGGCCAGATTGAAGGTCTTGCTAGACAAGCTGAGAGGAGGTGCATGGCCGTCGCCAGTTCGTGCCGTGAGGTGTCCTGTTAAGTCAGGCAACGATCGAGACCCACGCCTTTAGTTGCTACTGAGGAAATTTTCCGAGGGGCACTCTAAAGGGACTGCCGTCGACAAGACGGAGGAAGGAGTGGGCTACGGCAGGTCAGTATGCCCCGAATCCCCCGGGCTACACGCGAGCTGCAATGGCAGAGACAATGGGTTCCGACCCTGAAAAGGGAAGGCAATCTCTAAACTCTGCCTCAGTTTGGATTGAAGGCTGGAACCCGCCTTCATGAAAATGGAATGCCTAGTAATCGCGCGTCATCAACGCGCGGTGAATACGTCCCCGCTCCTTGCACACACCGCCCGTCGCTCCATCCGAGTGGGGTTTGGGTGAGGCGTGGTCTTCTTGGCTGCGTCGAATCTGAGTTCCGCAAGGAGGGAGAAGTCGTAACAAGGTGGCCGTAGGGGAACCTGCGGCCGGATCACCTCCT
>BA_Elba_MG_2_10-30cm
ATTCCGATTGATCCCGTCGGACCCCACTGCTATCGAGGTGGGACTAAGCCATGCTAGTCGAACGACCAGAACTACGTCTGGAAGTGGCATACTGCTCAGTAACACGTAGCTAACCTACCCTTAGGACGGAGATAACCCCGGGAAACTGGGGCTAATATACGATAGGTGTGATGTCCTGGAATGGTATCACGCCCAAAGGTGATAACAGCATGTTGTTATCATGCCTAAGGATGGGGCTGCGGCCGATCAGGCTGTTGGTGAGGTAACGGCTCACCAAACCTTTGACCGGTACGGGCCATGAGAGTGGTAGCCCGGAGATGGGCACTGAGACAAGGGCCCAGGCCCTACGGGGCGCAGCAGGCGCGAAACCTCCACAATTCACGAAAGTGAGATGGGGTTACCTCGAGTGCCGTCTGAAGAAGGCGGCTTTTCCATTGTGTAATGAGCCGTGGGAATAAGGGGGGGGCAAGACTGGTGTCAGCCGCCGCGGTAACACCAGCCCCCCGAGTGGTGGGGATGATTATTTGGCCTAAAGCGTCCGTAGCCGGCTCTGTAAATCTCTCGTTAAATCCAGCGTCCTAAGCGTTGGGCTGCGAGAGAGACTGCAGAGCTAGAGGGTGGGAGAGGTCAGCGGTATTCCTGGGGTAGGGGCGAAATCCATTGATCCCAGGAGGACCACCAGTGGCGAAGGCTGCTGACTAGAACACGCCTGACGGTGAGGGACGAAAGCTGGGGTAGCGAATCGGATTAGATACCCGAGTAGTCCCAGCCGTAAACGATGCAGGCTAGGTGTTGGGTCGGCCACGTGCCGCCTCAGTGCCACAGGGAAGCCATTAAGCCTGCCGCCTGGGGAGTACGGCCGCAAGGCTGAAACTTAAAGGAATTGGCGGGGGAGCACCACCAGGCGTGAAGCCTGCGGTTTAATTGGAGTCAACGCCGGGAACCTTACCGGGAGCGACAGCAGAGTGAAGGCCAGGTTGAAGGTCTTGCCGGACGAGCTGAGAGGAGGTGCATGGCCGTCGCCAGTTCGTGCCGTGAGGTGTCCTCTTAAGTGAGGTAACGAACGAGACCCGCGCCCCATGTTGCCATCAAATCCTAAAGGGATGTTGGGCACTCTTGGGGGACCGCAGCCGACAAGGCTGAGGAAGGTGCGGGCCACGGCAGGTCAGTATGCCCCGAATCTCCCGGGCCACACGCGGGCTGCAATGGTAGGGACAGAGGGCTGCAACCCCGAGAGGGGAAGCCAATCCCTAAACCCTACCTCAGTTGGGATCGAGGGCTGCAACCCGCCCTCGTGAACATGGAATGCCTAGTAACCGCCGGTCATCATCCGGCGGTGAATACGTCCCTGCTCCTTGCACACACTGCCCGTCGAACCACCCGAATGAGGTTTTGATGAGGCATGGTCGAAAGTTGGCCGTGTCGAATCTGGGCCTCGTAAGGAGGGTTAAGTCGTAACAAGGTGGCCGTAGGGGAACCTGCGGCCGGATCACCTCCT
>BA_Elba_MG_3_10-30cm
CTCCGGTTGATCCTGCCGGACCCCACTGCTATCGGGATAGGACTAAGACATGCTAGTCGAGCGCACTCAAGCCAATATGAGAGCGCGGCGCACAGCTCAGTAACACGTGGCTAACCTGCCCTCGGGACGGAGACAACCCCGGGAAACTGGGGCTAATCCCCGATAGGCGAAGAACTCTGGAATGAGTCTCCACCCAAAAGACGTTTACGTTATGCTCGTAGACACCGCCCAAGGATGGGGCCGCGTCCGATCAGGTTGTTGGTAAGGTAACGGCTCACCAAGCCTTTTACCGGTGCGGGCCGTGAGAGCGGGAGCCCGGAGATGGGCACTGAGACAAGGGCCCAGGCCCTACGGGGCGCAGCAGTCGCGAAAACTTTGCAATACACGAAAGTGTGACAGGGCTATCCCGAGTGCCATCCGCTGAGGAAGGCTTTTACCCAGTTTAGAACGCTGGGGGAATAAGGAGAGGGCAAGACTGGTGTCAGCCGCCGCGGTAATACCAGCTCTCCGAGTGGTGTGGATGTTTATTGGGCCTAAAGCATCCGTAGCTGGCTGGGCAAGTCCCCTGTTAAATCCACCGATTTAATCGTTGGAATGCGGGGGATACTGTTCGGCTAGGGGACGGGAGAGGCAGACGGTATTTTCGGGGTAGGGGTGAAATCCTATAATCCCGGGAAGACCACCAGTGGCGAAGGCTGTCTGCTAGAACGCGCCCGACGGTGAGGGATGAAAGCTGGGGGAGCGAACCGGATTAGATACCCGGGTAGTCCCAGCTGTAAACGATGCAGGCTAGGTGTTTGGGCGGCCACGTGCCGCTCTAGTGCCGCAGGGAAGCCGTTAAGCCTGCCGCCTGGGGAGTACGGCCGCAAGGCTGAAACTTAAAGGAATTGGCGGGGGAGCACCACAAGGGGTGAAGCTTGCGGTTTAATTGGAGTCAACGCCGGAAATCTCACCGGGAGCGACAGCAGTGTGAAGGCCAGATTAAAGGTCTTGCTGGACGAGCTGAGAGGAGGTGCATGGCCGTCGCCAGTTCGTGCCGTGAGGTGTCCTGTTAAGTCAGGCAACGATCGAGACCCGCGTCCTCTGTTGCTACTGGTCTTGTGCCAGGCACACTGAGGAGACCGCCGCTGATAAAGCGGAGGAAGGAGCGGGCCACGGCAGGTCAGTATGCCCCGAATCTCCCGGGCCACACGCGAGCTGCAATGGCAGGTACAATTGGTTCCAACCCCGAAAGGGGAAGGCAATCCCGAAAGCCTGCCGTAGTTGGGATCGAGGGTTGAAACCCATCCTCGTGAACATGGAATCCCTAGTAATCGCGTGTCACTAGCGCGCGGTGAATACGTCCCTGCTCCTTGCACACACCGCCCGTCGTTCCACCCGAGCGACTCTTGGATGAGGCGTGGTCTTTCTTGGCTGTGTCGAACCTGAGTTTTGTGAGGGGGGAAAAGTCGTAACAAGGTGGCCGTAGGGGAACCTGCGGCCGGATCACCTCCT
>BA_Elba_MG_4_10-30cm
ATACGGCTCAGTAACACGTGGTTAACCTACCCTCAGGACAGGGATAAACCCGGGAAACTGGGGCTAATATCTGATAGGCGAGGGCGCCTGGAATGGTTCTTCACCTAAAGGGCTTGAAAACATGCTTTCAAGTCGCCTGGGGATGGGGCCGCGTCCGATCAGGTTGTTGGTGAGGTAATGGCTCACCAAGCCTATAACCGGTACGGGCCGTGAGAGCGGTAGCCCGGAGATGGGTACTGAGACAAGGACCCAGGCCCTACGGGGCGCAGCAGTCGCGAAAACTCCGCAATGCACGAAAGTGTGACGGGGCTACCCCGAGTGCCGTCCGCTGAGGATGGCTTTTCCTTGGTGTAACTAGCCGGGGGAATAAGGAGAGGGCAAGACTGGTGTCAGCCGCCGCGGTAATACCAGCTCTCCGAGTGGTAGGGATGATTATTGGGCTTAAAGCGTCCGTAGCCGGCTTAGCAAGTCTCCTGTTAAATTCAGCGACCTAATCGTTGAGCCGCGGGAGATACTACTAGGCTAGGAGGCGGGAGAGGTCGACGGTACTTCCGGGGTAGGGGTGAAATCCTATAATCCCGGGAGGACCACCAGTGGCGAAGGCTGTCGACTAGAACGCGCTCGACGGTGAGGGACGAAAGCTGGGGGAGCGAACCGGATTAGATACCCGGGTAGTCCCAGCTGTAAACGATGCGGGCTAGGTGTTGGGGTGGCTACGAGCCACTTCAGTGCCGCAGGGAAGCCATTAAGCCCGCCGCCTGGGGAGTACGGCCGCAAGGCTGAAACTTAAAGGAATTGGCGGGGGAGCACCACAAGGCGTGAAGCTTGCGGTTCAATTGGAGTCAACGCCGGGAACCTTACCGGGGGCGACAGCAGGATGAATGCCAGATTGAAGGTCTTGCTGGACAAGCTGAGAGGAGGTGCATGGCCGTCGCCAGTTCGTGCCGTGAGGTGTCCTGTTAAGTCAGGCAACGATCGAGACCCGCGCCCCTAGTTGCTACTGAGGAAGTTTTCCAAGGGGCACTCTAGGGGGACTGCCGTCGACAAGACGGAGGAAGGAGCGGGCCACGGCAGGTCAGTATGCCCCGAATCCCCCGGGCTACACGCGAGCTGCAATGGCAGGGACAATGGGTTCCGACCTTGAGAAAGGAAGGCAATCTCTAAACCCTGCCTCAGTTTGGATCGAAGGCTGGAACCCGCCTTCGTGAAAATGGAATGCCTAGTAATCGCGCGTCATCATCGCGCGGTGAATACGTCCCCGCTCCTTGCACACACCGCCCGTCGCTCCATCCGAGTGGGGTTTGGGTGAGGCGTGGTCTTCTTGGCTGCGTCGAATCTGAGTTCCGCAAGGAGGGAGAAGTCGTAACAAGGTGGCCGTAGGGGAACCTGCGGCCGGATCACCTCCT
>BA_Elba_MG_5_30-45cm
TACACACGCCCGAGGATGGGGCTGCGGCCGATCAGGCAGTTGGTGAGGTAACGGCTCACCAAACCTTTGACCGGTACGGGCCGTGAGAGCGGTAGCCCGGAGATGGGCACTGAGACAAGGGCCCAGGCCCTACGGGGCGCAGCAGGCGCGAAACCTCCACAATGCACGCAAGTGCGATGGGGTTACCTCGAGTGCCGTCTGAAGAAGGCGGCTTTTCCATGGTGTAATGAGCCGTGGGAATAAGGGGGGGGCAAGACTGGTGTCAGCCGCCGCGGTAACACCAGCCCCCCGAGTGGTGGGGATGATTATTTGGCCTAAAGCGTCCGTAGCCGGCTCTGTAAATCTCTCGTTAAATCCAGCGTCCTAAGCGTTGGGCTGCGAGAGACACTGCAGAGCTAGAGGGTGGGAGAGGTCAGCGGTATTCCTGGGGTAGGGGCGAAATCCATTGATCCCAGGAGGACCACCAGTGGCGAAGGCTGCTGTCCAGAACACGCCTGACGGTCAGGGACGAAAGCTGGGGTAGCGAATCGGATTAGATACCCGAGTAGTCCCAGCCGTAAACGATGCAGGCTAGGTGTTGGGTCGGCCACGCGCCGCCTCAGTGCCACAGGGAAGCCATTAAGCCTGCCGCCTGGGGAGTACGGCCGCAAGGCTGAAACTTAAAGGAATTGGCGGGGGAGCACCACCAGGCGTGAAGCCTGCGGTTTAATTGGAGTCAACGCCGGGAACCTTACCGGGAGCGACAGCAGAGTGAAGGCCAGGTTGAAGGTCTTGCCGGACGAGCTGAGAGGAGGTGCATGGCCGTCGCCAGTTCGTGCCGTGAGGTGTCCTCTTAAGTGAGGTAACGAACGAGACCCGCGCCCCATGTTGCCAGCAAATCCTAAAGGGATGTTGGGCACTCTTGGGGGACCGCAGCCGACAAGGCTGAGGAAGGTGCGGGCAACGGCAGGTCAGTATGCCCCGAATCTCCCGGGCCACACGCGGGCTGCAATGGTAGTGACAGAGGGCTGCGACCCCGAGAGGGGGAGCCAATCCCTAAACACTACCTCAGTTGGGATCGAGGGCTGCAACCCGCCCTCGTGAACATGGAATGCCTAGTAACCGCCGGTCATCATCCGGCGGTGAATACGTCCCTGCTCCTTGCACACACTGCCCGTCGAACCACCCGAATGAGGTTTGGGTGAGGCATGGTCGAATGTTGGCCGTGTCGAACCTGGGCCTCGTAAGGAGGGTTAAGTCGTAACAAGGTGGCCGTAGGGGAACCTGCGGCCGGATCACCTCCT
>BA_Elba_MG_6_30-45cm
ACAGCTCAGTAACATGTGGCTAATCTGCCCTTAAGACAGAGATATCCCCGGGAAACTGGGGCTAATCTCTGATAGGTGTGGATTTCTGGAATGAATCTACACCCAAAGGGTGTCTGGATCATGCTTCGGACATCGCTTAAGGATGAGGCCGCAGCCGATCAGGTTGTTGGCGAGGTAACGGCTCACCAAGCCTATAACCGGTACGGGCCGCGAGAGCGGTAGCCCGGAGATGGACACTGAGACAAGGGTCCAGGCCCTACGGGGCGCAGCAGTTACGAAAACTCCGCAATGCACGAAAGTGTGACGGGGCTATCCCGAGTGCCGTCCGCTGAGGATGGCTTTTCTCCAGTGTAAGGAGCTGGAGGAATAAGGAGAGGGCAAGCCTGGTGTCAGCCGCCGCGGTAATACCAGCTCTCCGAGTGGTAGGGATGTTTATTGGGCTTAAAGCGTCCGTAGCCGGCTCAGAAAGTCCTCCGTTAAATCCAACGATTTAATCGTTGGACTGCGGGGGATACTCTTGGGCTTGGGGACGGGAGAGGTCGACGGTATTCTCGGAGTAGGGGTGAAATCCTATAATTCCGGGAGGACCACCAGTGGCGAAGGCTGTCGACTGGAACGAGCTCGACGGTGAGGGACGAAAGCTGGGGGAGCGAACCGGATTAGATACCCGGGTAGTCCCAGCTGTAAACGATGCGGGCTAGGTGTTAGAATGGCTACGAGCCATTCTAGTGCCGCAGCGAAGGCATTAAGCCCGCCGCCTGGGGAGTACGGCCGCAAGGCTGAAACTTAAAGGAATTGGCGGGAGAGCACCACAAGGCGTGAAGCGTGCGGTTTAATTGGAGTCAACACCGGAAACCTTACCGGGGGCGACAGCAGGTTGAAGGCCAGATTGACGATCTTGCCAGACAAGCTGAGAGGAGGTGCATGGCCGTCGCCAGTTCGTGCCGTGAGGTGTCCTGTTAAGTCAGGCAACGATCGAGACCCACGCTCCCTGTTGCTACTGAGGAAAGTTTCCAAGGGGCACTCTGGGAGGACTGCCGCCGACAAGGCGGAGGAAGGAGTGGGCCACGGCAGGTCAGTATGCCCCGAATCCCCCGGGCCACACGCGTGCTGCAATGGCCGGTACAATGAGTTCCTACCTCGTGAGAGGATGGCAATCCCTAAAACCGGTCGTAGTTTGGATCGAGGGCTGCAACCCGCCCTCGTGAA
>BA_Elba_MG_8_30-45cm
ATTCCGGTTGATCCTGCCGGACCTTACTGCTATCGGGTTGAGACTAAGCCATGCTAGTTGAGCGTTTTCAAGCTATGTTGAAAGCGCGGCAAACAGCTCACTAACACGTAGTTAACCTACCCTCAGGATGGAGATAACCCCTGGGAAACTGGGATTAATATCCAATAAATGATTATGCCTGGAATGGTTTATCATTGAAAGATCTGGAAACATGCTTTCAGTATCACCTGAGGATGGGACTGCGTCCGATCAGGTTGTTGGTGGGGTAACGGCCCACCAAGCCTATAACCGGTACGGGCCCTGAGAGGGGGAGCCCGGAGATGGAAACTGAGACAAGGTTCCAGGCCCTACGGGGCGCAGCAGGCGCGAAACCTCCACAATGCGCGAAAGCGTGATGGGGTTAGCCCGAGTGCTGTCCGAAAAGGATGGCTTTTCCGTGGTGTAAAAATCCATGGGAATAAAGGGGGGGCAAGACTGGTGTCAGCCGCCGCGGTAATACCAGCCCCCTGAGTGGTAAGGACGATTATTTGGCTTAAAGCGTCCGTAGCCGGCTTAGTGAGTCTTTTGTTAAATCCAATGATTCAATCATTGAACTGCAAGAGATACTGCTATGCTAGAGGACGGGAGAGGTCGACGGTAGTCCAGGGGTAGGGGTGAAATCCTATAATCCTTGGAGGACCACCAGTGGCGAAGGCGGTCGACTAGAACGTGCCTGACGGTGAGGGACGAAAGCTGGGGGAGCGAACCGGATTAGATACCCGGGTAGTCCCAGCTGTAAACGATGCGGGCTAGGTGTAGGGGTAGCTACGAGCTGCTCCTGTGCCGCAGAGAAATTGTTAAGCCCGCCGCCTGGGGAGTACGATCGCAAGATTGAAACTTAAAGGAATTGGCGGGGGAGCACCACAAGGGGTGAAGCCTGCGGTTTAATTGGAGTCAACGCCGGGAACCTTACCGGGGGCGACAGCAGGATGAAGGCCAGATTGAAGGTCTTGCCAGACAAGCTGAGAGGAGGTGCATGGCCGTCGACAGTTCGTGCCGTGAGGTGTCCTGTTAAGTCAGGCAACGAACGAGACCCCCACCATTA
>BA_Elba_MG_9_30-45cm
ATCCGGTTGATCCTGCCGGACCCTACTGCTATCGGGGTGGGACTAAGACATGCGAGTCGAGCGTCTCCAGCTATGGTGAGACGCGGCAGACGGCTCAGTAACACGTGGCTAACCTACCCTCAGGACAGGGACAAACCCGGGAAACTGGGGCTAATACCCGATAGGCGAGGGCGCCTGGAATGGTTCTTCACCCAAAGGGCTTTGGAAGCATGCTTTTCAAAGTCGCCTGGGGATGGGGCCGCGTCCGATCAGGTTGTTGGTGAGGTAACGGCTCACCAAGCCTATAACCGGTACGGGCCGTGAGAGCGGTAGCCCGGAGATGGGCACTGAGACAAGGGCCCAGGCCCTACGGGGCGCAGCAGTCGCGAAAACTCCGCAATGCACGAAAGTGTGACGGGGCTACCCCGAGTGCCGTCCGCTGAGGATGGCTTTTCCTCGGTGTAACTAGCCGGGGGAATAAGGAGAGGGCAAGACTGGTGTCAGCCGCCGCGGTAATACCAGCTCTCCGAGTGGTAGGGATGATTATTGGGCTTAAAGCGTCCGTAGCCGGCTTAGCAAGTCTCCTGTTAAATTCAGCGACCTAATCGTTGAGCCGCGGGAGATACTACTAGGCTAGGAGGCGGGAGAGGTCGACGGTACTTCTGGGGTAGGGGTGAAATCCTATAATCCCGGGAGGACCACCAGTGGCGAAGGCTGTCGACTAGAACGCGCTCGACGGTGAGGGACGAAAGCTGGGGGAGCGAACCGGATTAGATACCCGGGTAGTCCCAGCCGTAAACGATGCGGGCTAGGTGTTGGGGTGGCTACGAGCCACTTCAGTGCCGCAGGGAAGCCATTAAGCCCGCCGCCTGGGGAGTACGGCCGCAAGGCTGAAACTTAAAGGAATTGGCGGGGGAGCACCACAAGGCGTGAAGCTTGCGGTTCAATTGGAGTCAACGCCGGGAACCTTACCGGGGGCGACAGCAGGATGAATGCCAGATTGAAGGTCTTGCTGGACAAGCTGAGAGGAGGTGCATGGCCGTCGCCAGTTCGTGCCGTGAGGTGTCCTGTTAAGTCAGGCAACGATCGAGACCCGCGCCCCTAGTTGCTACTGAGGAAGTTTTCCAAGGGGCACTCTAGGGGGACTGCCGTCGACAAGACGGAGGAAGGAGCGGGCCACGGCAGGTCAGTATGCCCCGAATCCCCCGGGCTACACGCGAGCTGCAATGGCAGGGACAATGGGTTCCCACCTTGAAAAAGGGAGGCAATCCCTAAACCCTGCCTCAGTTGGGATCGAGGGCTGCAACTCGCCCTCGTGAACATGGAATGCCTAGTAATCGCGCGTCATCATCGCGCGGTGAATACGTCCCCGCTCCTTGCACACACCGCCCGTCGCTCCATCCGAGTGGGGTTTGGGTGAGGCGTGGTCTTCTTGGCTGCGTCGAATCTGAGTTCCGCAAGGAGGGAGAAGTCGTAACAAGGTGGCCGTAGGGGAACCTGCGGCCGGATCACCTCCT
>BA_Elba_MG_10_30-45cm
CTCCGGTTGATCCTGCCGGACCCCACTGCTATTGGGATAGGACTAAGACATGCTAGTCAAGCGCTCGCCAGCCAAAGGTGAGTGCGGCGTACAGCTCAGTAACACGTGGCTAACCTGCCCTTGGGACGGGGACACCCCCGGGAAACTGGGGTTAATCCCCGATAGGCGGAGAACTCTGGAATGAATCTCCACCCAAAAGGCACTTGCGCTATGCTCGTAGGTGTTGCCCAAGGATGGGGCCGCGACCGATCAGGTTGTTGGTGAGGTAATGGCTCACCAAGCCTTTTACCGGTGCGGGCCGTGAGAGCGGGAGCCCGGAGATGGGCACTGAGACAAGGGCCCAGGCCCTACGGGGCGCAGCAGTCGCGAAAACTTTGCAATACACGAAAGTGTGACAGGGCTATCCCGAGTGCCATCCGCTGAGGAAGGCTTTTACCCAGTGTAGAAAGCTGGGAGAATAAGGAGAGGGCAAGACTGGTGTCAGCCGCCGCGGTAATACCAGCTCTCCGAGTGGTGTGGATGTTTATTGGGCCTAAAGCATCCGTAGCTGGCTGGGCAAGTCCCCTGTTAAATCCACCGATTTAATCGTTGGAATGCGGGGGATACTGTTCGGCTAGGGGACGGGAGAGGCAGACGGTATTTTCGGGGTAGGGGTGAAATCCTATAATCCCGGGAAGACCACCAGTGGCGAAGGCTGTCTGCTAGAACGCGCCCGACGGTGAGGGATGAAAGCTGGGGGAGCGAACCGGATTAGATACCCGGGTAGTCCCAGCTGTAAACGATGCAGGCTAGGTGTTTGGGCGGCCACGTGCCGCTCTAGTGCCGCAGGGAAGCCGTTAAGCCTGCCGCCTGGGGAGTACGGCCGCAAGGCTGAAACTTAAAGGAATTGGCGGGGGAGCACCACAAGGGGTGAAGCTTGCGGTTTAATTGGAGTCAACGCCGGAAATCTCACCGGGAGCGACAGCAGTGTGAAGGCCAGATTAAAGGTCTTGCTGGACGAGCTGAGAGGAGGTGCATGGCCGTCGCCAGTTCGTGCCGTGAGGTGTCCTGTTAAGTCAGGCAACGATCGAGACCCGCGTCCTCTGTTGCTACTGGTCTTGTGCCAGGCACACTGAGGAGACCGCCGTTGATAAAGCGGAGGAAGGAGCGGGCCACGGCAGGTCAGTATGCCCCGAATCTCCCGGGCTACACGCGAGCTGCAATGGCAGGTACAATTGGTCCCGACCTCGAAAGGGGAAGGCAATCCCGAAAGCTTGCCGTAGTTGGGATCGAGGGTTGAAACCCACCCTCGTGAACATGGAATCCCTAGTAATCGCGTGTCACTAGCGCGCGGTGAATACGTCCCTGCTCCTTGCACACACCGCCCGTCGTTCCACCCGAGCGACTCTTGGATGAGGCGTGGTCATTATTGGCTGTGTCGAATCTGAGTTTTGTGAGGGGGGAAAAGTCGTAACAAGGTGGCCGTAGGGGAACCTGCGGCCGGATCACCTCCT
>BA_Elba_MG_11_2-10cm
CAGCTCTCCGAGTGGTAGGGATGATTATTGGGCTTAAAGCGTCCGTAGCCGGCTTAGCAAGTTTCCTGTTAAACTCAGCGACTCAATCGTTGACCCGCGGGAAATACTACTAGGCTAGGAGGCGGGAGAGGTCGACGGTACTTCTGGGGTAGGGGTGAAATCCTATAATCCCGGGAGGACCACCAGTGGCGAAGGCTGTCGACTAGAACGCGCTCGACGGTGAGGGACGAAAGCTGGGGGAGCGAACCGGATTAGATACCCGGGTAGTCCCAGCCGTAAACGATGCGGGCTAGGTGTTGGGATGGCTACGTGCCATTTCAGTGCCGCAGGGAAGCCATTAAGCCCGCCGCCTGGGGAGTACGGCCGCAAGGCTGAAACTTAAAGGAATTGGCGGGGGAGCACCACAAGGCGTGAAGCTTGCGGTTCAATTGGAGTCAACGCCGGGAACCTTACCGGGGGCGACAGCAGAATGAAGGCCAGATTGAAGGTCTTGCTAGACAAGCTGAGAGGAGGTGCATGGCCGTCGCCAGTTCGTGCCGTGAGGTGTCCTGTTAAGTCAGGCAACGATCGAGACCCACGCCTTTAGTTGCTACTGAGGAAGTTTTCCGAGGGGCACTCTAAAGGGACTGCCGTCGACAAGACGGAGGAAGGAGCGGGCCACGGCAGGTCAGTATGCCCCGAATCCCCCGGGCTACACGCGAGCTGCAATGGCAGGGACAATGGGTTCCGACCCTGAAAAGGGAAGGCAATCTCTAAACCCTGCCTCAGTTTGGATCGAAGGCTGGAACCCGCCTTCGTGAAAATGGAATGCCTAGTAATCGCGCGTCATCATCGCGCGGTGAATACGTCCCCGCTCCTTGCACACACCGCCCGTCGCTCCATCCGAGTGGGGTTTGGGTGAGGCGTGGTCTTATTGGCTGCGTCGAATCTGAGTTCCGCAAGGAGGGAGAAGTCGTAACAAGGTGGCCGTAGGGGAACCTGCGGCCGGATCACCTCCT
>BA_Elba_MG_12_10-30cm
CTCCGGTTGATCCTGCCGGACCCCACTGCTATCGGGATAGGACTAAGACATGCTAGTCAAGCGCTCGCCAGCCAAAGGTGAGTGCGGCGTACAGCTCAGTAACACGTGGCTAACCTGCCCTTGGGACGGGGACACCCCCGGGAAACTGGGGTTAATCCCCGATAGGCGGAGAACTCTGGAATGAATCTCCACCCAAAAGGCACTTGCGCTATGCTCGTAGGTGTTGCCCAAGGATGGGGCCGCGACCGATCAGGTTGTTGGTGAGGTAATGGCTCACCAAGCCTTTTACCGGTGCGGGCCGTGAGAGCGGGAGCCCGGAGATGGGCACTGAGACAAGGGCCCAGGCCCTACGGGGCGCAGCAGTCGCGAAAACTTTGCAATACACGAAAGTGTGACAGGGCTATCCCGAGTGCCATCCGCTGAGGAAGGCTTTTACCCAGTTTAGAAAGCTGGGAGAATAAGGAGAGGGCAAGACTGGTGTCAGCCGCCGCGGTAATACCAGCTCTCCGAGTGGTGTGGATGTTTATTGGGCCTAAAGCATCCGTAGCTTGCTGAACAAGTCCTCTGTTAAACCCAACGAATTAATCGTTGGAGTGCGGGGGATACTGCTCGGCTTGGGGGCGAGAGAGGCAGACGGTATTTTCGGGGTAGGAGTGAAATCCTATAATCCCGGGAAGACCACCAGTGGCGAAGGCTGTCTGCTAGAACGCGCCCGACGGTGAGGGATGAAAGCTGGGGGAGCGAACCGGATTAGATACCCGGGTAGTCCCAGCTGTAAACGATGCAGGCTAGGTGTTTGGGCGGCCACGTGCCGCTCTAGTGCCGCAGGGAAGCCGTTAAGCCTGCCGCCTGGGGAGTACGGCCGCAAGGCTGAAACTTAAAGGAATTGGCGGGGGAGCACCACAAGGGGTGAAGCTTGCGGTTTAATTGGAGTCAACGCCGGAAATCTCACCGGGAGCGACAGCAGTGTGAAGGCCAGATTAAAGGTCTTGCTGGACGAGCTGAGAGGAGGTGCATGGCCGTCGCCAGTTCGTGCCGTGAGGTGTCCTGTTAAGTCAGGCAACGATCGAGACCCGCGTCCTCTGTTGCTACTGGTCTTGTGCCAGGCACACTGAGGAGACCGCCGTTGATAAAGCGGAGGAAGGAGCGGGCCACGGCAGGTCAGTATGCCCCGAATCTCCCGGGCTACACGCGAGCTGCAATGGCAGGTACAATTGGTCCCGACCTCGAAAGGGGAAGGCAATCCCGAAAGCCTGCCGTAGTTGGGATCGAGGGTTGAAACTCACCCTCGTGAACATGGAATCCCTAGTAATCGCGTGTCACTAGCGCGCGGTGAATACGTCCCTGCTCCTTGCACACACCGCCCGTCGTTCCACCCGAGCGACTCTTGGATGAGGCGTGGTCTTTCTTGGCTGTGTCGAATCTGAGTTTCGTGAGGGGGGAAAAGTCGTAACAAGGTGGCCGTAGGGGAACCTGCGGCCGGATCACCTCCT
>BA_Elba_MG_13_10-30cm
ATCCGGTTGATCCTGCCGGACCCTACTGCTATCGGGGTGGGACTAAGACATGCGAGTCGAGCGTCTCTAGCTATGTTGAGACGCGGCAGACGGCTCAGTAACACGTGGCTAACCTACCCTCAGGACAGGGACAAACCCGGGAAACTGGGGCTAATACCCGATAGGCGAGGGCGCCTGGAATGGTTCTTCACCCAAAGGGCTTTGGAAGCATGCTTTTCAGAGTCGCCTGGGGATGGGGCCGCGTCCGATCAGGTTGTTGGTGGGGTAACGGCTCACCAAGCCTATAACCGGTACGGGCCGTGAGAGCGGTAGCCCGGAGATGGGCACTGAGACAAGGGCCCAGGCCCTACGGGGCGCAGCAGTCGCGAAAACTCCGCAATGCACGAAAGTGTGACGGGGCTACCCCGAGTGCCGTCCGCTGAGGATGGCTTTTCCTTGGTGTAACTAGCCGGGGGAATAAGGAGAGGGCAAGACTGGTGTCAGCCGCCGCGGTAATACCAGCTCTCCGAGTGGTAGGGATGATTATTGGGCTTAAAGCGTCCGTAGCCGGCTTAGCAAGTCTCCTGTTAAATTCAGCGACCTAATCGTTGAGCCGCGGGAGATACTACTAGGCTAGGAGGCGGGAGAGGTCGACGGTACTTCTGGGGTAGGGGTGAAATCCTATAATCCCGGGAGGACCACCAGTGGCGAAGGCTGTCGACTAGAACGCGCTCGACGGTGAGGGACGAAAGCTGGGGGAGCGAACCGGATTAGATACCCGGGTAGTCCCAGCTGTAAACGATGCGGGCTAGGTGTTGGGGTGGCTACGAGCCACTTCAGTGCCGCAGGGAAGCCATTAAGCCCGCCGCCTGGGGAGTACGGCCGCAAGGCTGAAACTTAAAGGAATTGGCGGGGGAGCACCACAAGGCGTGAAGCTTGCGGTTCAATTGGAGTCAACGCCGGGAACCTTACCGGGGGCGACAGCAGGATGAAGGCCAGATTGAAGGTCTTGCTGGACAAGCTGAGAGGAGGTGCATGGCCGTCGCCAGTTCGTGCCGTGAGGTGTCCTGTTAAGTCAGGCAACGATCGAGACCCGCGCCCCTAGTTGCTACTGAGGAAGTTTTCCAAGGGGCACTCTAGGGGGACTGCCGTCGACAAGACGGAGGAAGGAGCGGGCCACGGCAGGTCAGTATGCCCCGAATCCCCCGGGCTACACGCGAGCTGCAATGGCAGGGACAATGGGTTCCGACCCTGAAAAGGGGAGGCAATCTCTAAACCCTGCCTCAGTTGGGATCGAAGGCTGGAACCCGCCTTCGTGAACATGGAATGCCTAGTAATCGCGCGTCATCATCGCGCGGTGAATACGTCCCCGCTCCTTGCACACACCGCCCGTCGCTCCATCCGAGTGGGGTTTGGGTGAGGCGTGGTCTTCTTGGCTGCGTCGAATCTGAGTTCCGCAAGGAGGGAGAAGTCGTAACAAGGTGGCCGTAGGGGAACCTGCGGCCGGATCACCTCCT
>BA_Elba_MG_14_30-45cm
CTCCGGTTGATCCTGCCGGACCCCACTGCTATCGGGATAGGACTAAGACATGCTAGTCAAGCGCTCGCCAGCCAAAGGTGAGTGCGGCGTACAGCTCAGTAACACGTGGCTAACCTGCCCTTGGGACGGGGACACCCCCGGGAAACTGGGGTTAATCCCCGATAGGCGGAGAACTCTGGAATGAATCTCCACCCAAAAGGCACTTGCGCTATGCTCGTAGGTGTTGCCCAAGGATGGGGCCGCGACCGATCAGGTTGTTGGTGAGGTAATGGCTCACCAAGCCTTTTACCGGTGCGGGCCGTGAGAGCGGGAGCCCGGAGATGGGCACTGAGACAAGGGCCCAGGCCCTACGGGGCGCAGCAGTCGCGAAAACTTTGCAATACACGAAAGTGTGACAGGGCTATCCCGAGTGCCATCCGCTGAGGAAGGCTTTTACCCAGTGTAGAAAGCTGGGAGAATAAGGAGAGGGCAAGACTGGTGTCAGCCGCCGCGGTAATACCAGCTCTCCGAGTGGTGTGGATGTTTATTGGGCCTAAAGCATCCGTAGCTGGCTGGGCAAGTCCCCTGTTAAATCCACCGATTTAATCGTTGGAGTGCGGGGGATACTGTTCGGCTAGGGGACGGGAGAGGCAGACGGTATTTTCGGGGTAGGGGTGAAATCCTATAATCCCGGGAAGACCACCAGTGGCGAAGGCTGTCTGCTAGAACGCGCCCGACGGTGAGGGATGAAAGCTGGGGGAGCGAACCGGATTAGATACCCGGGTAGTCCCAGCTGTAAACGATGCAGGCTAGGTGTTTGGGCGGCCACGTGCCGCTCTAGTGCCGCAGGGAAGCCGTTAAGCCTGCCGCCTGGGGAGTACGGCCGCAAGGCTGAAACTTAAAGGAATTGGCGGGGGAGCACCACAAGGGGTGAAGCTTGCGGTTTAATTGGAGTCAACGCCGGAAATCTCACCGGGAGCGACAGCAGTGTGAAGGCCAGATTAAAGGTCTTGCTGGACGAGCTGAGAGGAGGTGCATGGCCGTCGCCAGTTCGTGCCGTGAGGTGTCCTGTTAAGTCAGGCAACGATCGAGACCCGCGTCCTCTGTTGCTACTGGTCTTGTGCCAGGCACACTGAGGAGACCGCCGCTGATAAAGCGGAGGAAGGAGCGGGCCACGGCAGGTCAGTATGCCCCGAATCTCCCGGGCCACACGCGAGCTGCAATGGCAGGTACAATGGGTTCCGACCTCGAAAGGGGAAGGCAATCCCGAAAGCCTGCCGTAGTTGGGATCGAGGGTTGAAACCCACCCTCGTGAACATGGAATCCCTAGTAATCGCGTGTCACTAGCGCGCGGTGAATACGTCCCTGCTCCTTGCACACACCGCCCGTCGTTCCACCCGAGCGACTCTTGGATGAGGCGTGGTCTTTCTTGGCTGTGTCGAATCTGAGTTTCGTGAGGGGGGAAAAGTCGTAACAAGGTGGCCGTAGGGGAACCTGCGGCCGGATCACCTCCT
>BA_Elba_MG_15_30-45cm
CGCGAAACCTCCACAATTCACGAAAGTGAGATGGGGTTACCTCGAGTGCCGCCTGAAGAAGGCGGCTTTTCCATGGTGTAATGAGCCGTGGGAATAAGGGGGGGGCAAGACTGGTGTCAGCCGCCGCGGTAACACCAGCTCCCCGAGTGGTGGGGATGATTATTTGGCCTAAAGCGTCCGTAGCCGGCTAAGTAAATTTCTCGTTAAATCCAACGTCCTAAGCGTTGGGCTGCGAGAAAGACTGCTCAGCTAGAGGGTGGGAGAGGTCAGCGGTATTCCTGGGGTAGGGGCGAAATCCATTGATCCCAGGAGGACCACCAGTGGCGAAGGCTGCTGACTAGAACACGCCTGACGGTGAGGGACGAAAGCTGGGGTAGCGAATCGGATTAGATACCCGAGTAGTCCCAGCCGTAAACGATGCAGGCTAGGTGTTGGGTCGGCCATGGGCCGCCTCAGTGCCACAGGGAAGCCATTAAGCCTGCCGCCTGGGGAGTACGGCCGCAAGGCTGAAACTTAAAGGAATTGGCGGGGGAGCACCACCAGGCGTGAAGCCTGCGGTTTAATTGGAGTCAACGCCGGGAACCTTACCGGGAGCGACAGCAGAGTGAAGGCCAGGTTGAAGGTCTTGCCAGACGAGCTGAGAGGAGGTGCATGGCCGTCGCCAGTTCGTGCCGTGAGGTGTCCTCTTAAGTGAGGTAACGAACGAGACCCGCGCCCCATGTTGCCAGCAAATCCTAAAGGGATGTTGGGCACTCTTGGGGGACCGCAGCCGACAAGGCTGAGGAAGGTGCGGGCAACGGCAGGTCAGTATGCCCCGAATCTCCCGGGCCACACGCGGGCTGCAATGGTAGTGACAGAGGGCTGCGACCCCGAGAGGGGGAGCCAATCCCTAAACACTACCTCAGTTGGGATCGAGGGCTGCAACCCGCCCTCGTGAACATGGAATGCCTAGTAACCGCCGGTCATCATCCGGCGGTGAATACGTCCCTGCTCCTTGCACACACTGCCCGTCGAACCACCCGAATGAGGTTTGGGTGAGGCATGGTCGAAAGTTGGCCGTGTCGAACCTGGGCCTCGTAAGGAGGGTTAAGTCGTAACAAGGTGGCCGTAGGGGAACCTGCGGCCGGATCACCTCCT
>BA_Elba_MG_16_30-45cm
ATCCGGTTGATCCTGCCGGACCCTACTGCTATCGGGGTGGGACTAAGACATGCGAGTCGAGCGTCTCTAGTTATGGTGAGACGCGGCAGACGGCTCAGTAACACGTGGCTAACCTACCCTCAGGACAGGGACAAACCCGGGAAACTGGGGCTAATACCCGATAGGCGAGGGCGCCTGGAATGGTTCTTCACCCAAAGGGCTTTGGAAGCATGCTTTTCAGAGTCGCCTGGGGATGGGGCCGCGTCCGATCAGGTTGTTGGTGAGGTAACGGCTCACCAAGCCTATAACCGGTACGGGCCGTGAGAGCGGTAGCCCGGAGATGGGCACTGAGACAAGGGCCCAGGCCCTACGGGGCGCAGCAGTCGCGAAAACTCCGCAATGCACGAAAGTGTGACGGGGCTACCCCGAGTGCCGTCCGCTGAGGATGGCTTTTCCTTGGTGTAACTAGCCGGGGGAATAAGGAGAGGGCAAGACTGGTGTCAGCCGCCGCGGTAATACCAGCTCTCCGAGTGGTAGGGATGATTATTGGGCTTAAAGCGTCCGTAGCCGGCTTAGCAAGTCTCCTGTTAAATTCAGCGACCTAATCGTTGAGCCGCGGGAGATACTACTAGGCTAGGAGGCGGGAGAGGTCGACGGTACTTCCGGGGTAGGGGTGAAATCCTATAATCCCGGGAGGACCACCAGTGGCGAAGGCTGTCGACTAGAACGCGCTCGACGGTGAGGGACGAAAGCTGGGGGAGCGAACCGGATTAGATACCCGGGTAGTCCCAGCCGTAAACGATGCGGGCTAGGTGTTGGGGTGGCTACGTGCCACTTCAGTGCCGCAGGGAAGCCATTAAGCCCGCCGCCTGGGGAGTACGGCCGCAAGGCTGAAACTTAAAGGAATTGGCGGGGGAGCACCACAAGGCGTGAAGCTTGCGGTTCAATTGGAGTCAACGCCGGGAACCTTACCGGGGGCGACAGCAGGATGAATGCCAGATTGAAGGTCTTGCTGGACAAGCTGAGAGGAGGTGCATGGCCGTCGCCAGTTCGTGCCGTGAGGTGTCCTGTTAAGTCAGGCAACGATCGAGACCCGCGCCCCTAGTTGCTACTGAGGAAGTTTTCCAAGGGGCACTCTAGAGGGACTGCCGTCGACAAGACGGAGGAAGGAGCGGGCCACGGCAGGTCAGTATGCCCCGAATCCCCCGGGCTACACGCGAGCTGCAATGGCAGGGACAATGGGTTCCGACCCTGAAAAGGGAAGGCAATCTCTAAACCCTGCCTCAGTTTGGATCGAAGGCTGGAACCCGCCTTCGTGAAAATGGAATGCCTAGTAATCGCGCGTCATCATCGCGCGGTGAATACGTCCCCGCTCCTTGCACACACCGCCCGTCGCTCCATCCGAGTGGGGTTTGGGTGAGGCGTGGTCTTCTTGGCTGCGTCGAATCTGAGTTCCGCAAGGAGGGAGAAGTCGTAACAAGGTGGCCGTAGGGGAACCTGCGGCCGGATCACCTCCT
>BA_Elba_MG_17_10-30cm
ATTCCGATTGATCCCGTCGGACCCCACTGCTATCGAGGTGGGACTAAGCCATGCTAGTCGAACGAACAGAACTATGTCTGTAAGTGGCACACTGCTCAGTAACACGTAGCTAACCTACCCTTAGGACGTTAATAACCCCGAGAAATTGGGACTAATGAACGATAGATGTATTGTCCTGGAATGGTATTACGTCCAAAGGTATCTCATACATGTTTGGTATACGCCTAAGGATGGGGCTGCGGCCGATCAGGCTGTTGGTGAGGTAATGGCTCACCAAACCTTTGACCGGTACGGGCCATGAGAGTGGTAGCCCGGAGATGGACACTGAGACAAGGGTCCAGGCCCTACGGGGCGCAGCAGGCGCGAAACCTCCTCAATGCACTAACGTGCGAAGGGGTTACCTCGAGTGCTATCTGAAGAAGGTGGCTTTTCCTTGGTCTAAAAAGCCTTGGGAATAAGAGGGGGGCAAGACTGGTGTCAGCCGCCGCGGTAACACCAGCCCCTCGAGTGGTGGGGATGATTATTTGGCCTAAAGCGTCCGTAGCCGGCCGGGTAAGTTTCCCGTTAAATCCAGCGTCTCAAGCGTTGGACTGCGGGAAATACTGCCTGGCTAGAGAGTGGGAGAGGTCAGCGGTATTCTGGGGGTAGGGGCGAAATCCTTTGATCCCCGGAGGACCACCAGTGGCGAAGGCTGCTGACTAGAACACGTCTGACGGTCAGGGACGAAAGCTGGGGTAGCGAACCGGATTAGATACCCGGGTAGTCCCAGCCGTAAACGATGCATGCTAGGTGTTGGGTTGGCCACGAGCCACCCCAGTGCCGCAGGGAAGCCGTTAAGCATGCCGCCTGGGAAGTACGATCGCAAGGTTGAAACTTAAAGGAATTGGCGGGGGAGCACCACCAGGCGTGAAGCCTGCGGTTTAATTGGAGTCAACGCCGGGAACCTTACCGGGAGCGACAGCAGAGTGAAGGCCAGATTGAAGATCTTGCCAGACGAGCTGAGAGGAGGTGCATGGCCGTCGCCAGTTCGTGCCGTGAGGTGTTCTCTTAAGTGAGATAACGAACGAGACCCGTGCCGCATGTTGCCACCAGAATCTAAAGGATTGCTGAGCACTCTTGTGGGACCGCAGCCGATAAGGCTGAGGAAGGTACGGGCAACGGCAGGTCAGTATGCCCCGAATCTCCCGGGCCACACGCGGGCTGCAATGGTACAGACAGAGGGCTGCCACCCCGAGAGGGGGAGCCAATCCCTAAACTGTACCTCAGTTGGGATCGAGGGTTGCAACCCACCCTCGTGAACATGGAATGCCTAGTAACCGCCAGTCATCATCTGGCGGTGAATACGTCCCTGCTCCTTGCACACACTGCCCGTCGAACCACCCGAATGAGGTTTGGGTGAGGAATGGTCGAATGTTGGCCGTTTCGAACCTGGGCCTCGTAAGGAGGGTTAAGTCGTAACAAGGTGGCCGTAGGGGAACCTGCGGCCGGATCACCTCCT
>BA_Elba_MG_18_10-30cm
GATGCCTGGAATGGTTTCTCGCTCAAAGGATCTTGGAAACATGCTTCCAAGTCGCCTGAGGATGGGGCCGCGTCCGATCAGGTTGTTGGTGAGGTAACGGCTCACCAAGCCTATAACCGGTACGGGCCATGAGAGTGGTAGCCCGGAGATGGGTACTGAGACAAGGACCCAGGCCCTACGGGGCGCAGCAGTCGCGAAAACTCCGCAATGCACGCAAGTGTGACGGGGCTACCCCGAGTGCCGTCCGCTGAGGATGGCTTTTCCTTGGTGTAACTAGCCAGGGGAATAAGGAGAGGGCAAGACTGGTGTCAGCCGCCGCGGTAATACCAGCTCTCCGAGTGGTAGGGATGATTATTGGGCTTAAAGCGTCCGTAGCCGGCTTAGCAAGTCTCCTGTTAAATTCAGCGACCTAATCGTTGAGCCGCGGGAGATACTACTAGGCTAGGAGGCGGGAGAGGTCGACGGTATTTCCGGGGTAGGGGTGAAATCCTATAATCCCGGGAGGACCACCAGTGGCGAAGGCTGTCGACTAGAACGCGCTCGACGGTGAGGGACGAAAGCTGGGGGAGCGAACCGGATTAGATACCCGGGTAGTCCCAGCCGTAAACGATGCGGGCTAGGTGTTGGGATGGCTACGTGCCATTTCAGTGCCGCAGGGAAGCCATTAAGCCCGCCGCCTGGGGAGTACGGCCGCAAGGCTGAAACTTAAAGGAATTGGCGGGGGAGCACCACAAGGCGTGAAGCTTGCGGTTCAATTGGAGTCAACGCCGGGAACCTTACCGGGGGCGACAGCAGGATGAATGCCAGATTGAAGGTCTTGCTGGACAAGCTGAGAGGAGGTGCATGGCCGTCGCCAGTTCGTGCCGTGAGGTGTCCTGTTAAGTCAGGCAACGATCGAGACCCGCAACCTTAGTTGCAACCTCCGCGGAATCCGCAGAGGGCACACTACGGGAACTGCCGCCGACAAGGCGGAGGAAGGAGCGGGCCACGGCAGGTCAGTATGCCCCGAATCCCCCGGGCTACACGCGAGCTGCAATGGCAGGGACAATGGGTTCCGACCCTGAAAAGGGAAGGCAATCTCTAAACCCTGCCTCAGTTTGGATCGAAGGCTGGAACCCGCCTTCGTGAAAATGGAATGCCTAGTAATCGCGCGTCATCAACGCGCGGTGAATACGTCCCCGCTCCTTGCACACACCGCCCGTCGCTCCATCCGAGTGGGGTTTGGGTGAGGCGTGGTCTTTTTGGCTGCGTCGAATCTGAGTTCCGCAAGGAGGGTGAAGTCGTAACAAGGTGGCCGTAGGGGAACCTGCGGCCGGATCACCTCCT
>BA_Elba_MG_19_30-45cm
ATCCGGTTGATCCTGCCGGACCCTACTGCTATCGGGGTGGGACTAAGACATGCGAGTCGAGCGTCTCCAGCTATGGTGAGACGCGGCAGACGGCTCAGTAACACGTGGCTAACCTACCCTCAGGACAGGGACAAACCCGGGAAACTGGGGCTAATACCCGATAGGCGAGGGCGCCTGGAATGGTTCTTCACCCAAAGGGCTTTGGAAGCATACTTTTCAAAGTCGCCTGGGGATGGGGCCGCGTCCGATCAGGTTGTTGGTGAGGTAACGGCTCACCAAGCCTATAACCGGTACGGGCCGTGAGAGCGGTAGCCCGGAGATGGGCACTGAGACAAGGGCCCAGGCCCTACGGGGCGCAGCAGGCGCGAAACCTCCACAATGCACGCAAGTGCGATGGGGCTACCTCGAGTGCCGCCTGAAGAAGGCGGCTTTTCCACGGTGTAATGAGCCGTGGGAATAAGAGGGGGGCAAGACTGGTGTCAGCCGCCGCGGTAACACCAGCCCCTCGAGTGGTGGGGATGATTATTTGGCCTAAAGCGTCCGTAGCCGGCTCGGTAAATCTCTCGTTAAATCCAGCGTCCTAAGCGTTGGGCTGCGAGGGAGACTGCCGAGCTAGAGGGTGGGAAAGGTCAGCGGTATTTCTGGGGTAGGGGCGAAATCCATTGATCCCAGGAGGACCACCAGTGGCGAAGGCTGCTGACTAGAACACGCCTGACGGTGAGGGACGAAAGCTGGGGTAGCGAATCGGATTAGATACCCGAGTAGTCCCAGCCGTAAACGATGCAGGCTAGGTGTTGGGTTGGCCACGCGCCAGCTCAGTGCCACAGGGAAGCCATTAAGCCTGCCGCCTGGGGAGTACGGCCGCAAGGCTGAAACTTAAAGGAATTGGCGGGGGAGCACCACCAGGCGTGAAGCCTGCGGTTTAATTGGAGTCAACGCCGGGAACCTTACCGGGAGCGACAGCAGAGTGAAGGCCAGGTTGAAGGTCTTGCCGGACGAGCTGAGAGGAGGTGCATGGCCGTCGCCAGTTCGTGCCGTGAGGTGTCCTCTTAAGTGAGGTAACGAACGAGACCCGCGCCCCATGTTGCCATCAAATCCTAAAGGGATGTTGGGCACTCTTGGGGGACCGCAGCCGACAAGGCTGAGGAAGGTGCGGGCAACGGCAGGTCAGTATGCCCCGAATCTCCCGGGCCACACGCGGGCTGCAATGGTAGGGACAGAGGGCTGCAACCCCGAGAGGGGAAGCCAATCCCTAAATCCTACCTCAGTTGGGATCGAGGGCTGCAACCCGCCCTCGTGAACATGGAATGCCTAGTAACCGCGCGTCATCATCGCGCGGTGAATACGTCCCCGCTCCTTGCACACACTGCCCGTCGAACCACCCAAATGGAGCTTGGATGAGGCGTGGTCGAAAGTTGGCCGCTTCGAATTTGGGCTCCGTGAGGAGGGTTAAGTCGTAACAAGGTGGCCGTAGGGGAACCTGCGGCCGGATCACCTCCT
>BA_Elba_MG_20_30-45cm
ATCCGGTTGATCCTGCCGGACCTTACTGCTATCGGGGTGGGACTAAGACATGCGAGTCGAGCGTCTCTAGCTATGTTGAGACGCGGCAGACGGCTCAGTAACACGTGGCTAACCTACCCTCAGGACGAGGACAAACCCGGGAAACTGGGGCTAATACTCGATAGGTGAGGATATCTGGAATGATTTCTCACTCAAAGGGCCTTGGAAACATGCTTCCAATGTCGCCTGGGGATGGGGCCGCGTCCGATCAGGTTGTTGGTGAGGTAACGGCTCACCAAGCCTATAACCGGTACGGGCCGTGAGAGCGGTAGCCCGGAGATGGGTACTGAGACAAGGACCCAGGCCCTACGGGGCGCAGCAGTCGCGAAAACTCCGCAATGCACGAAAGTGTGACGGGGCTACCCCGAGTGCCGTCCGCTGAGGATGGCTTTTCCTCGGTGTAACTAGCCGGGGGAATAAGGAGAGGGCAAGACTGGTGTCAGCCGCCGCGGTAATACCAGCTCTCCGAGTGGTAGGGATGATTATTGGGCTTAAAGCGTCCGTAGCCGGCTTAGCAAGTCTCCTGTTAAATTCAGCGACCTAATCGTTGAGCCGCGGGAGATACTACTAGGCTAGGAGGCGGGAGAGGTCGACGGTACTTCTGGGGTAGGGGTGAAATCCTATAATCCCGGGAGGACCACCAGTGGCGAAGGCTGTCGACTAGAACGCGCTCGACGGTGAGGGACGAAAGCTGGGGGAGCGAACCGGATTAGATACCCGGGTAGTCCCAGCCGTAAACGATGCGGGCTAGGTGTTGGGGTGGCTACGTGCCACTTCAGTGCCGCAGGGAAGCCATTAAGCCCGCCGCCTGGGGAGTACGGCCGCAAGGCTGAAACTTAAAGGAATTGGCGGGGGAGCACCACAAGGCGTGAAGCTTGCGGTTCAATTGGAGTCAACGCCGGGAACCTTACCGGGGGCGACAGCAGGATGAAGGCCAGATTGAAGGTCTTGCTGGACAAGCTGAGAGGAGGTGCATGGCCGTCGCCAGTTCGTGCCGTGAGGTGTCCTGTTAAGTCAGGCAACGATCGAGACCCACGCCTCTAGTTGCTACTGAGGAAGTTTTCCAAGGGGCACTCTAGAGGGACTGCCGTCGACAAGACGGAGGAAGGAGTGGGCCACGGCAGGTCAGTATGCCCCGAATCCCCCGGGCTACACGCGAGCTGCAATGGCAGGGACAATGGGTTCCGACCCTGAAAAGGGGAGGCAATCCTTAAACCCTGCCTCAGTTGGGATCGAAGGCTGGAACCCGCCTTCGTGAACATGGAATGCCTAGTAATCGCGCGTCATCATCGCGCGGTGAATACGTCCCCGCTCCTTGCACACACCGCCCGTCGCTCCATCCGAGTGGGGTTTGGGTGAGGCGTGGTCTTCTTGGCTGCGTCGAATCTGAGTTCCGCAAGGAGGGAGAAGTCGTAACAAGGTGGCCGTAGGGGAACCTGCGGCCGGATCACCTCCT
>BA_Elba_MG_21_30-45cm
CTCCGGTTGATCCTGCCGGACCCCACTGCTATTGGGATAGGACTAAGACATGCTAGTCAAGCGCTCGCCAGCCAAAGGTGAGTGCGGCGTACAGCTCAGTAACACGTGGCTAACCTGCCCTTGGGACGGGGACACCCCCGGGAAACTGGGGTTAATCCCCGATAGGCGGAGAACTCTGGAATGAATCTCCACCCAAAAGGCACTTGCGCTATGCTCGTAGGTGTTGCCCAAGGATGGGGCCGCGACCGATCAGGTTGTTGGTGAGGTAATGGCTCACCAAGCCTTTTACCGGTGCGGGCCGTGAGAGCGGGAGCCCGGAGATGGGCACTGAGACAAGGGCCCAGGCCCTACGGGGCGCAGCAGTCGCGAAAACTTTGCAATACACGAAAGTGTGACAGGGCTATCCCGAGTGCCATCCGCTGAGGAAGGCTTTTACCCAGTTTAGAACGCTGGGGGAATAAGGAGAGGGCAAGACTGGTGTCAGCCGCCGCGGTAATACCAGCTCTCCGAGTGGTGTGGATGTTTATTGGGCCTAAAGCATCCGTAGCTGGCTGGGCAAGTCCCCTGTTAAATCCACCGATTTAATCGTTGGAATGCGGGGGATACTGTTCGGCTAGGGGACGGGAGAGGCAGACGGTATTTTCGGGGTAGGGGTGAAATCCTATAATCCCGGGAAGACCACCAGTGGCGAAGGCTGTCTGCTAGAACGCGCCCGACGGTGAGGGATGAAAGCTGGGGGAGCGAACCGGATTAGATACCCGGGTAGTCCCAGCTGTAAACGATGCAGGCTAGGTGTTTGGGCGGCCACGTGCCGCTCTAGTGCCGCAGGGAAGCCGTTAAGCCTGCCGCCTGGGGAGTACGGTCGCAAGGCTGAAACTTAAAGGAATTGGCGGGGGAGCACCACAAGGGGTGAAGCTTGCGGTTTAATTGGAGTCAACGCCGGAAATCTCACCGGGAGCGACAGCAGTGTGAAGGCCAGATTAAAGGTCTTGCTGGACGAGCTGAGAGGAGGTGCATGGCCGTCGCCAGTTCGTGCCGTGAGGTGTCCTGTTAAGTCAGGCAACGATCGAGACCCGCGTCCTCTGTTGCTACTGGTCTTGTGCCAGGCACACTGAGGAGACCGCCGTTGATAAAGCGGAGGAAGGAGCGGGCCACGGCAGGTCAGTATGCCCCGAATCTCCCGGGCTACACGCGAGCTGCAATGGCAGGTACAATTGGTCCCGACCTCGAAAGGGGAAGGCAATCCCGAAAGCTTGCCGTAGTTGGGATCGAGGGTTGAAACCCACCCTCGTGAACATGGAATCCCTAGTAATCGCGTGTCACTAGCGCGCGGTGAATACGTCCCTGCTCCTTGCACACACCGCCCGTCGTTCCACCCGAGCGACTCTTGGATGAGGCGTGGTCATTATTGGCTGTGTCGAATCTGAGTTTCGTGAGGGGGGAAAAGTCGTAACAAGGTGGCCGTAGGGGAACCTGCGGCCGGATCACCTCCT
>BA_Elba_Bin_1_30-45cm
CTCCGGTTGATCCTGCCGGACCCCACTGCTATTGGGATAGGACTAAGACATGCTAGTCAA
GCGCTCGCCAGCCAAAGGTGAGTGCGGCGTACAGCTCAGTAACACGTGGCTAACCTGCCC
TTGGGACGGGGACACCCCCGGGAAACTGGGGTTAATCCCCGATAGGCGGAGAACTCTGGA
ATGAATCTCCACCCAAAAGGCACTTGCGCTATGCTCGTAGGTGTTGCCCAAGGATGGGGC
CGCGACCGATCAGGTTGTTGGTGAGGTAATGGCTCACCAAGCCTTTTACCGGTGCGGGCC
GTGAGAGCGGGAGCCCGGAGATGGGCACTGAGACAAGGGCCCAGGCCCTACGGGGCGCAG
CAGTCGCGAAAACTTTGCAATACACGAAAGTGTGACAGGGCTATCCCGAGTGCCATCCGC
TGAGGAAGGCTTTTACCCAGTTTAGAACGCTGGGGGAATAAGGAGAGGGCAAGACTGGTG
TCAGCCGCCGCGGTAATACCAGCTCTCCGAGTGGTGTGGATGTTTATTGGGCCTAAAGCA
TCCGTAGCTGGCTGGGCAAGTCCCCTGTTAAATCCACCGATTTAATCGTTGGAATGCGGG
GGATACTGTTCGGCTAGGGGACGGGAGAGGCAGACGGTATTTTCGGGGTAGGGGTGAAAT
CCTATAATCCCGGGAAGACCACCAGTGGCGAAGGCTGTCTGCTAGAACGCGCCCGACGGT
GAGGGATGAAAGCTGGGGGAGCGAACCGGATTAGATACCCGGGTAGTCCCAGCTGTAAAC
GATGCAGGCTAGGTGTTTGGGCGGCCACGTGCCGCTCTAGTGCCGCAGGGAAGCCGTTAA
GCCTGCCGCCTGGGGAGTACGGTCGCAAGGCTGAAACTTAAAGGAATTGGCGGGGGAGCA
CCACAAGGGGTGAAGCTTGCGGTTTAATTGGAGTCAACGCCGGAAATCTCACCGGGAGCG
ACAGCAGTGTGAAGGCCAGATTAAAGGTCTTGCTGGACGAGCTGAGAGGAGGTGCATGGC
CGTCGCCAGTTCGTGCCGTGAGGTGTCCTGTTAAGTCAGGCAACGATCGAGACCCGCGTC
CTCTGTTGCTACTGGTCTTGTGCCAGGCACACTGAGGAGACCGCCGTTGATAAAGCGGAG
GAAGGAGCGGGCCACGGCAGGTCAGTATGCCCCGAATCTCCCGGGCTACACGCGAGCTGC
AATGGCAGGTACAATTGGTCCCGACCTCGAAAGGGGAAGGCAATCCCGAAAGCTTGCCGT
AGTTGGGATCGAGGGTTGAAACCCACCCTCGTGAACATGGAATCCCTAGTAATCGCGTGT
CACTAGCGCGCGGTGAATACGTCCCTGCTCCTTGCACACACCGCCCGTCGTTCCACCCGA
GCGACTCTTGGATGAGGCGTGGTCATTATTGGCTGTGTCGAATCTGAGTTTCGTGAGGGG
GGAAAAGTCGTAACAAGGTGGCCGTAGGGGAACCTGCGGCCGGATCACCTCCT
>BA_Elba_Bin_2_10-30cm
CTCCGGTTGATCCTGCCGGACCCCACTGCTATCGGGATAGGACTAAGACATGCTAGTCAA
GCGCTCGCCAGCTAAAGGTGAGTGCGGCGCACAGCTCAGTAACACGTGGCTAACCTGCCC
TTGGGACGGGGACACCCCCGGGAAACTGGGGTTAATCCCCGATAGGCGGAGAACTCTGGA
ATGAATCTCTACCCAAAGGGCGCTTGCGCTATGCTCGCAGGTGTTGCCCAAGGATGGGGC
CGCGACCGATCAGGTTGTTGGTGAGGTAATGGCTCACCAAGCCTTTTACCGGTGCGGGCC
GTGAGAGCGGGAGCCCGGAGATGGGCACTGAGACAAGGGCCCAGGCCCTACGGGGCGCAG
CAGTCGCGAAAACTTTGCAATACACGAAAGTGTGACAGGGCTATCCCGAGTGCCATCCGC
TGAGGAAGGCTTTTACCCAGTTTAGAAAGCTGGGAGAATAAGGAGAGGGCAAGACTGGTG
TCAGCCGCCGCGGTAATACCAGCTCTCCGAGTGGTGTGGATGTTTATTGGGCCTAAAGCA
TCCGTAGCTGGCTGGGCAAGTCCCCTGTTAAATCCACCGATTTAATCGTTGGAATGCGGG
GGATACTGTTCGGCTAGGGGACGGGAGAGGCAGACGGTATTTTCGGGGTAGGGGTGAAAT
CCTATAATCCCGGGAAGACCACCAGTGGCGAAGGCTGTCTGCTAGAACGCGCCCGACGGT
GAGGGATGAAAGCTGGGGGAGCGAACCGGATTAGATACCCGGGTAGTCCCAGCTGTAAAC
GATGCAGGCTAGGTGTTTGGGCGGCCACGTGCCGCTCTAGTGCCGCAGGGAAGCCGTTAA
GCCTGCCGCCTGGGGAGTACGGCCGCAAGGCTGAAACTTAAAGGAATTGGCGGGGGAGCA
CCACAAGGGGTGAAGCTTGCGGTTTAATTGGAGTCAACGCCGGAAATCTCACCGGGAGCG
ACAGCAGTGTGAAGGCCAGATTAAAGGTCTTGCTGGACGAGCTGAGAGGAGGTGCATGGC
CGTCGCCAGTTCGTGCCGTGAGGTGTCCTGTTAAGTCAGGCAACGATCGAGACCCGCGTC
CTCTGTTGCTACTGGTCTTGTGCCAGGCACACTGAGGAGACCGCCGCTGATAAAGCGGAG
GAAGGAGCGGGCCACGGCAGGTCAGTATGCCCCGAATCTCCCGGGCCACACGCGAGCTGC
AATGGCAGGTACAATTGGTTCCAACCCCGAAAGGGGAAGGCAATCCCGAAAGCCTGCCGT
AGTTGGGATCGAGGGTTGAAACCCATCCTCGTGAACATGGAATCCCTAGTAATCGCGTGT
CACTAGCGCGCGGTGAATACGTCCCTGCTCCTTGCACACACCGCCCGTCGTTCCACCCGA
GCGACTCTTGGATGAGGCGTGGTCTTTCTTGGCTGTGTCGAATCTGAGTTTTGTGAGGGG
GGAAAAGTCGTAACAAGGTGGCCGTAGGGGAACCTGCGGCCGGATCACCTCCT
>BA_Elba_Bin_3_10-30cm
CTCCGGTTGATCCTGCCGGACCCCACTGCTATTGGGATAGGACTAAGACATGCTAGTCAA
GCGCTCGCCAGCCAAAGGTGAGTGCGGCGTACAGCTCAGTAACACGTGGCTAACCTGCCC
TTGGGACGGGGACACCCCCGGGAAACTGGGGTTAATCCCCGATAGGCGGAGAACTCTGGA
ATGAATCTCCACCCAAAAGGCACTTGCGCTATGCTCGTAGGTGTTGCCCAAGGATGGGGC
CGCGACCGATCAGGTTGTTGGTGAGGTAATGGCTCACCAAGCCTTTTACCGGTGCGGGCC
GTGAGAGCGGGAGCCCGGAGATGGGCACTGAGACAAGGGCCCAGGCCCTACGGGGCGCAG
CAGTCGCGAAAACTTTGCAATACACGAAAGTGTGACAGGGCTATCCCGAGTGCCATCCGC
TGAGGAAGGCTTTTACCCAGTTTAGAACGCTGGGGGAATAAGGAGAGGGCAAGACTGGTG
TCAGCCGCCGCGGTAATACCAGCTCTCCGAGTGGTGTGGATGTTTATTGGGCCTAAAGCA
TCCGTAGCTGGCTGGGCAAGTCCCCTGTTAAATCCACCGATTTAATCGTTGGAATGCGGG
GGATACTGTTCGGCTAGGGGACGGGAGAGGCAGACGGTATTTTCGGGGTAGGGGTGAAAT
CCTATAATCCCGGGAAGACCACCAGTGGCGAAGGCTGTCTGCTAGAACGCGCCCGACGGT
GAGGGATGAAAGCTGGGGGAGCGAACCGGATTAGATACCCGGGTAGTCCCAGCTGTAAAC
GATGCAGGCTAGGTGTTTGGGCGGCCACGTGCCGCTCTAGTGCCGCAGGGAAGCCGTTAA
GCCTGCCGCCTGGGGAGTACGGCCGCAAGGCTGAAACTTAAAGGAATTGGCGGGGGAGCA
CCACAAGGGGTGAAGCTTGCGGTTTAATTGGAGTCAACGCCGGAAATCTCACCGGGAGCG
ACAGCAGTGTGAAGGCCAGATTAAAGGTCTTGCTGGACGAGCTGAGAGGAGGTGCATGGC
CGTCGCCAGTTCGTGCCGTGAGGTGTCCTGTTAAGTCAGGCAACGATCGAGACCCGCGTC
CTCTGTTGCTACTGGTCTTGTGCCAGGCACACTGAGGAGACCGCCGCTGATAAAGCGGAG
GAAGGAGCGGGCCACGGCAGGTCAGTATGCCCCGAATCTCCCGGGCTACACGCGAGCTGC
AATGGCAGGTACAATTGGTCCCGACCTCGAAAGGGGAAGGCAATCCCGAAAGCTTGCCGT
AGTTGGGATCGAGGGTTGAAACCCACCCTCGTGAACATGGAATCCCTAGTAATCGCGTGT
CACTAGCGCGCGGTGAATACG
>BA_Elba_Bin_4_30-45cm
CTCCGGTTGATCCTGCCGGACCCCACTGCTATTGGGATAGGACTAAGACATGCTAGTCAA
GCGCTCGCCAGCCAAAGGTGAGTGCGGCGTACAGCTCAGTAACACGTGGCTAACCTGCCC
TTGGGACGGGGACACCCCCGGGAAACTGGGGTTAATCCCCGATAGGCGGAGAACTCTGGA
ATGAATCTCCACCCAAAAGGCACTTGCGCTATGCTCGTAGGTGTTGCCCAAGGATGGGGC
CGCGACCGATCAGGTTGTTGGTGAGGTAATGGCTCACCAAGCCTTTTACCGGTGCGGGCC
GTGAGAGCGGGAGCCCGGAGATGGGCACTGAGACAAGGGCCCAGGCCCTACGGGGCGCAG
CAGTCGCGAAAACTTTGCAATACACGAAAGTGTGACAGGGCTATCCCGAGTGCCATCCGC
TGAGGAAGGCTTTTACCCAGTGTAGAAAGCTGGGAGAATAAGGAGAGGGCAAGACTGGTG
TCAGCCGCCGCGGTAATACCAGCTCTCCGAGTGGTGTGGATGTTTATTGGGCCTAAAGCA
TCCGTAGCTGGCTGGGCAAGTCCCCTGTTAAATCCACCGATTTAATCGTTGGAGTGCGGG
GGATACTGTTCGGCTAGGGGACGGGAGAGGCAGACGGTATTTTCGGGGTAGGGGTGAAAT
CCTATAATCCCGGGAAGACCACCAGTGGCGAAGGCTGTCTGCTAGAACGCGCCCGACGGT
GAGGGATGAAAGCTGGGGGAGCGAACCGGATTAGATACCCGGGTAGTCCCAGCTGTAAAC
GATGCAGGCTAGGTGTTTGGGCGGCCACGTGCCGCTCTAGTGCCGCAGGGAAGCCGTTAA
GCCTGCCGCCTGGGGAGTACGGCCGCAAGGCTGAAACTTAAAGGAATTGGCGGGGGAGCA
CCACAAGGGGTGAAGCTTGCGGTTTAATTGGAGTCAACGCCGGAAATCTCACCGGGAGCG
ACAGCAGTGTGAAGGCCAGATTAAAGGTCTTGCTGGACGAGCTGAGAGGAGGTGCATGGC
CGTCGCCAGTTCGTGCCGTGAGGTGTCCTGTTAAGTCAGGCAACGATCGAGACCCGCGTC
CTCTGTTGCTACTGGTCTTGTGCCAGGCACACTGAGGAGACCGCCGTTGATAAAGCGGAG
GAAGGAGCGGGCCACGGCAGGTCAGTATGCCCCGAATCTCCCGGGCTACACGCGAGCTGC
AATGGCAGGTACAATTGGTCCCGACCTCGAAAGGGGAAGGCAATCCCGAAAGCTTGCCGT
AGTTGGGATCGAGGGTTGAAACCCACCCTCGTGAACATGGAATCCCTAGTAATCGCGTGT
CACTAGCGCGCGGTGAATACGTCCCTGCTCCTTGCACACACCGCCCGTCGTTCCACCCGA
GCGACTCTTGGATGAGGCGTGGTCATTATTGGCTGTGTCGAATCTGAGTTTTGTGAGGGG
GGAAAAGTCGTAACAAGGTGGCCGTAGGGGAACCTGCGGCCGGATCACCTCCT
